# Machine learning-based prediction of prolonged length of stay in older patients with type 2 diabetes mellitus and cardiovascular disease

**DOI:** 10.3389/fcvm.2026.1903579

**Published:** 2026-08-05

**Authors:** Yixia Zuo, Jianfei Chen, Jie Wang, Zheng Zhu, Longao Huang, Qi Deng, Yan Li, Liping You, Jun Gu

**Affiliations:** Department of Cardiology, Affiliated Banan Hospital of Chongqing Medical University, Chongqing, China

**Keywords:** cardiovascular disease, machine learning, prolonged length of stay, type 2 diabetes mellitus, XGBoost

## Abstract

**Background:**

Older patients with type 2 diabetes mellitus (T2DM) and cardiovascular disease (CVD) frequently experience prolonged length of stay (PLOS). This condition increases healthcare burden and worsens prognosis. However, no predictive model specifically addresses PLOS in this high-risk multimorbid population.

**Methods:**

This single-center retrospective study included hospitalized older T2DM-CVD patients. PLOS was defined as hospital stay exceeding the 75th percentile of the training set population. Potential predictors were selected via LASSO regression. Eight machine learning (ML) models were developed to predict PLOS risk. Model performance was evaluated using receiver operating characteristic curves, calibration curves, and decision curve analysis. SHAP analysis was employed for model interpretability.

**Results:**

A total of 27,629 patients were included. The XGBoost model achieved the highest training AUC (0.819) and demonstrated competitive predictive performance in both the internal (AUC = 0.753) and time-based external (AUC = 0.728) validation sets. However, its performance advantage over simpler models such as logistic regression was modest in validation, and XGBoost showed some degree of overfitting (AUC drop of 0.066 from training to validation). Although logistic regression showed comparable validation performance with less overfitting, XGBoost was selected as the final model for its ability to capture complex nonlinear interactions and provide SHAP-based interpretability, with the understanding that further external validation is needed. Key predictors included cerebral infarction, white blood cell count, anemia, pulse rate, the glycated hemoglobin to high-density lipoprotein cholesterol ratio (GHR), and osteoporosis. Most continuous variables showed nonlinear associations with PLOS risk.

**Conclusions:**

The XGBoost-based model effectively predicts PLOS risk in older T2DM-CVD patients. This tool shows promise for early identification of high-risk individuals and optimization of medical resource allocation within our institutional setting. However, further prospective and multi-center validation studies are required before clinical adoption.

## Introduction

Type 2 diabetes mellitus (T2DM) and cardiovascular disease (CVD) represent the two most prevalent chronic conditions among the older population and frequently coexist, creating complex clinical scenarios (1). Globally, T2DM affects more than 537 million adults, with the highest prevalence observed among individuals aged 65 years and older (2). Epidemiological data indicate that diabetes increases the risk of CVD by two- to four-fold, and among older patients with diabetes, CVD-related mortality is approximately 32% higher compared to those with normoglycemia (3, 4). An individual participant data meta-analysis comprising 82,723 older adults further confirmed that T2DM is associated with a 44% increased risk of CVD events or all-cause mortality (HR: 1.44, 95% CI: 1.40–1.49) (5). The prognosis of older patients with T2DM complicated by CVD is particularly poor, with hospitalization burden, complication rates, and mortality significantly higher than those in patients with either condition alone (6).

Length of stay is a key indicator of healthcare quality and patient prognosis, and shortening hospital stay helps alleviate the burden on medical systems (7). For older patients with T2DM complicated by CVD, prolonged length of stay (PLOS) not only signifies excessive consumption of medical resources but also often reflects potential areas for optimization in clinical management. Studies have shown that cardiovascular comorbidities are important determinants of PLOS in T2DM patients (8, 9).

Although previous studies have separately investigated risk factors for PLOS in patients with either T2DM or CVD, research specifically targeting the distinct multimorbid population of “older T2DM with concurrent CVD” remains very limited (10, 11). To date, no predictive model for PLOS has been specifically developed for this population. Furthermore, existing prediction tools for T2DM-related complications have predominantly relied on conventional logistic regression, which may inadequately capture the complex nonlinear interactions among clinical variables in this multimorbid older cohort (12–15). While several ML studies have been applied to T2DM patients with cardiovascular conditions, their primary outcome was 30-day readmission rather than PLOS, leaving the prediction of prolonged hospitalization specifically in this population unexplored (2). Therefore, the present study aims to develop and validate multiple machine learning (ML)-based predictive models for PLOS risk in older patients with T2DM complicated by CVD using real-world data, thereby providing an evidence-based tool for early identification of high-risk individuals and optimization of medical resource allocation.

## Methods

### Study design and data source

This retrospective study initially enrolled 49,049 hospitalized patients diagnosed with T2DM complicated by CVD at the Affiliated Banan Hospital of Chongqing Medical University between January 2018 and June 2025. The exclusion criteria were as follows: (1) age <65 years; (2) in-hospital death; (3) concomitant malignancy; and (4) hospital stay <24 h. Patients who died during hospitalization were excluded because the clinical objective of predicting PLOS is most relevant for patients who are expected to be discharged. For patients with in-hospital death, length of stay is confounded by the competing event of mortality. Predicting PLOS in this population would have limited clinical utility. Furthermore, these patients represented a small proportion of the overall cohort, and their exclusion is unlikely to substantially affect the generalizability of the model to the broader population of older T2DM-CVD patients. After applying these criteria, a total of 25,101 eligible patients were included from this period. An additional time-based external validation cohort comprising 3,825 patients hospitalized between July 2025 and December 2025 was subsequently enrolled using the same inclusion and exclusion criteria, yielding 2,528 eligible patients. Thus, a total of 27,629 eligible patients were included in the final analysis. The internal cohort was randomly divided into a training set (*n* = 17,570) and an internal validation set (*n* = 7,531) at a 7:3 ratio, and the 2,528 patients from the subsequent period were designated as the time-based external validation set. A detailed flowchart of the patient selection and exclusion process is provided in Supplementary Figure 1. The study was conducted in accordance with the Declaration of Helsinki and received approval from the Institutional Ethics Committee (Ethics number: BNLL-KY-2025-086). Owing to its retrospective design, the requirement for informed consent was waived. Furthermore, the development and reporting of the prediction models adhered to the Transparent Reporting of a Multivariable Prediction Model for Individual Prognosis or Diagnosis (TRIPOD) statement and its artificial intelligence extension, TRIPOD-AI (16).

### Data collection

Clinical data of the patients were extracted from the hospital's electronic medical record (EMR) system. All collected variables are summarized in Supplementary Table S1 and primarily fall into three categories: demographic information (age, sex, smoking history, drinking history), comorbidities [anemia, chronic obstructive pulmonary disease (COPD), cerebral infarction, osteoporosis, hypokalemia, chronic kidney disease (CKD)], and vital signs [systolic blood pressure (SBP), diastolic blood pressure (DBP), pulse rate]. In addition, a range of laboratory parameters and derived composite indices were recorded. The laboratory parameters included total cholesterol (TC), triglyceride (TG), creatinine (Cr), uric acid (UA), low-density lipoprotein cholesterol (LDL-C), high-density lipoprotein cholesterol (HDL-C), hemoglobin, glycated hemoglobin (HbA1c), fasting glucose (FG), white blood cell (WBC) count, and platelet (PLT) count. The composite indices comprised the triglyceride-glucose index (TyG), total cholesterol to high-density lipoprotein cholesterol ratio (TC/HDL-C), uric acid to high-density lipoprotein cholesterol ratio (UHR), atherogenic index of plasma (AIP), non-high-density lipoprotein cholesterol to high-density lipoprotein cholesterol ratio (NHHR), glycated hemoglobin to high-density lipoprotein cholesterol ratio (GHR), uric acid to creatinine ratio (UA/Cr), and stress hyperglycemia ratio (SHR).

For variables with a missing rate below 30% (Supplementary Tables S2–S4), multiple imputation was performed using the “mice” package in R (17). Missingness was assumed to be missing at random (MAR), as the probability of missingness was not systematically related to the unobserved values after accounting for observed covariates. The imputation procedure was performed exclusively within the training set to prevent information leakage, and the established imputation model was subsequently applied to both the internal validation set and the time-based external validation set. For continuous variables, predictive mean matching (PMM) was applied; for binary categorical variables, logistic regression was used; and for categorical variables with more than two categories, multinomial logistic regression was applied. To assess the stability of the imputation results, we examined the variability of imputed values across the five datasets and found that the standard deviations of the imputed values were consistently below 5% of their means, indicating minimal variability. Accordingly, we used the first imputed dataset for model training and evaluation, a practical approach recommended when imputation variability is low. To further assess the robustness of this approach, we evaluated the stability of model performance across the five imputed datasets. All composite indices were calculated after the imputation procedure was completed.

All predictor variables were measured or ascertained at admission or within the first 24 h of hospitalization, ensuring that the model can be applied at the intended clinical decision point (i.e., early after admission). Specifically, demographic characteristics and comorbidities were derived from the admission record and medical history; vital signs were recorded at admission; and laboratory parameters were obtained from the initial blood tests performed upon admission. Composite indices were calculated using these admission-based laboratory values.

### Definition of PLOS

The primary outcome of this study was to predict the presence of PLOS in older patients with T2DM complicated by CVD. Length of stay was defined as the interval from the day of admission to the day of discharge. Given the lack of a standardized definition for PLOS, it was defined as exceeding the 75th percentile of the training set population, in accordance with previous relevant studies (18–21). The 75th percentile threshold was calculated exclusively within the training set to prevent information leakage, and this threshold was then applied unchanged to both the internal validation set and the time-based external validation set. Accordingly, PLOS was operationalized as a length of stay of 12 days or more in this study.

### ML models development

To select predictors for model development, the least absolute shrinkage and selection operator (LASSO) regression was applied to all candidate variables in the training set (22). LASSO performs continuous shrinkage and automatic variable selection by penalizing the absolute size of regression coefficients, which helps mitigate overfitting and multicollinearity while preserving potentially important predictors regardless of their univariate associations. The optimal penalty parameter *λ* was determined via 10-fold cross-validation using the one-standard-error rule (*λ*_1_se), and variables with non-zero coefficients at this *λ* value were retained as final predictors. Based on the selected predictors, eight ML models were developed to predict the risk of PLOS in older patients with T2DM complicated by CVD. These models included artificial neural network (ANN), support vector machine (SVM), decision tree (DT), random forest (RF), gradient boosting decision tree (GBDT), light gradient boosting machine (LightGBM), extreme gradient boosting (XGBoost), and logistic regression (LR). All models were implemented using Python (version 3.12.3) with the scikit-learn (version 1.4.2), xgboost (version 2.0.3), and lightgbm (version 4.3.0) libraries. A random seed (random_state = 42) was set for all models to ensure reproducibility. For models sensitive to feature scaling (SVM and ANN), all continuous variables were standardized to zero mean and unit variance using StandardScaler; for tree-based models (DT, RF, GBDT, LightGBM, and XGBoost), no scaling was applied as these models are scale-invariant. Categorical variables were encoded using one-hot encoding. Hyperparameter tuning was performed using a grid search combined with 5-fold cross-validation exclusively within the training set to prevent information leakage. The optimal hyperparameters for each model were selected based on the highest cross-validated AUC. For XGBoost and LightGBM, early stopping with 50 rounds was applied to prevent overfitting. The final hyperparameters for each model are summarized in Supplementary Table S5.

The final model was selected using a pre-specified hierarchical multi-criteria process. Model performance was primarily evaluated using the area under the receiver operating characteristic (ROC) curve (AUC), supplemented by additional metrics including sensitivity, specificity, Youden's index, and F1 score. Among these, AUC was considered the most critical indicator of model performance. 95% CIs for all performance metrics were estimated via bootstrap with 1,000 resamples. To formally compare the discriminative performance between models, pairwise DeLong tests were performed between XGBoost and each of the other seven models in both the training and validation sets. For each model, the optimal threshold maximizing the Youden index was determined within the training set and then applied unchanged to both the internal and external validation sets to compute sensitivity, specificity, and F1 score. In addition, calibration curves were plotted and decision curve analysis (DCA) was performed to assess the agreement between model predictions and clinical practice, as well as the net clinical benefit. To quantitatively evaluate model calibration, we further calculated the Brier score, calibration slope, and calibration intercept. 95% CIs for all calibration metrics were estimated via bootstrap with 1,000 resamples. The Brier score measures the overall accuracy of probabilistic predictions, with values ranging from 0 (perfect calibration) to 0.25 (non-informative for a 50% event rate). The calibration slope and intercept assess the agreement between predicted probabilities and observed outcomes, with an ideal slope of 1.0 and an ideal intercept of 0. For the calibration intercept, a positive value indicates systematic underestimation of risk (predicted probabilities are too low), while a negative value indicates systematic overestimation of risk (predicted probabilities are too high). No additional class-balancing procedures were applied, except for the scale_pos_weight parameter in XGBoost, which was set to the imbalance ratio (minority/majority) to account for the modest class imbalance (1:2.71). This is a built-in weighting mechanism within the XGBoost algorithm rather than an external resampling or balancing procedure. No oversampling, undersampling, or synthetic data generation techniques were used.

The hierarchical criterion was defined as follows: (1) discriminative performance [AUC and precision-recall AUC (PR-AUC)] in the validation sets, with greater emphasis on validation than training performance to avoid overfitting; (2) calibration quality (Brier score, calibration slope, and calibration intercept); and (3) model interpretability and clinical utility. This multi-criteria approach was adopted to avoid over-reliance on any single metric and to ensure that the selected model balances performance with generalizability. No single metric dominated the selection when performance differences among models were modest; instead, the multi-criteria process was applied to balance performance, calibration, and interpretability.

### Model interpretation

To enhance the interpretability of the optimal model, the SHapley Additive exPlanation (SHAP) method was employed to quantify and visualize the contribution of each feature to the prediction outcome (23). SHAP values were calculated for each patient in the entire cohort, yielding the marginal effect of each feature on the outcome prediction. The SHAP analysis not only ranked feature importance but also revealed the well-balanced relationship between individual feature values and predicted outcomes. It is important to note, however, that SHAP values explain the behavior of the fitted model and reflect predictive associations; they do not establish causal relationships between predictors and PLOS.

### Statistical analysis

All statistical analyses and graphical presentations were performed using R software (version 4.3.3) and Python software (version 3.12.3). Categorical variables were presented as frequencies (percentages) and compared between groups using the chi-square test. Continuous variables were handled according to their distribution: those following a normal distribution were expressed as mean ± standard deviation and compared using Student's *t*-test, whereas non-normally distributed variables were summarized as median (25th percentile, 75th percentile) and compared using the Mann-–Whitney *U*-test. All tests were two-sided, and a *p*-value <0.05 was considered statistically significant. To assess multicollinearity among predictors, the variance inflation factor (VIF) and its reciprocal (tolerance) were calculated. Furthermore, restricted cubic spline (RCS) regression was employed to explore potential nonlinear relationships between continuous variables and PLOS (24). To evaluate the robustness of our PLOS definition, we performed sensitivity analyses using alternative thresholds: the 90th percentile of the training set distribution and a fixed cutoff of 14 days. For each alternative definition, the final selected model was retrained in the training set using the same hyperparameter tuning procedure, and performance was evaluated in the internal validation set and the time-based external validation set. This approach ensures that the sensitivity analysis assesses generalizability to unseen patients rather than merely reflecting training-set overoptimism.

## Results

### Study population characteristics

A total of 25,101 older patients with T2DM complicated by CVD were included from the internal cohort (January 2018 to June 2025), of whom 6,682 (26.62%) experienced PLOS. All participants in the internal cohort were randomly assigned to a training set (*n* = 17,570) and an internal validation set (*n* = 7,531) at a ratio of 7:3 (Table 1). The median age of the internal cohort was 74 years, and female patients accounted for 53.82% (13,509 cases). The proportion of patients with smoking history was 26.29% (6,598 cases). The proportion of patients with drinking history was 19.28% (4,839 cases). Regarding comorbidities, the prevalence rates of anemia, COPD, cerebral infarction, osteoporosis, hypokalemia, and CKD were 13.64%, 10.37%, 24.92%, 14.87%, 13.38%, and 12.21%, respectively. An additional time-based external validation set (July 2025 to December 2025) included 2,528 eligible patients, whose baseline characteristics are summarized in Supplementary Table S6. Missing data in all three sets were handled using multiple imputation, with detailed procedures documented in Supplementary Tables S7–S9. For the vast majority of variables, no statistically significant differences were observed before and after imputation (*P* > 0.05).

**Table 1:** Baseline characteristics of participants in the training and internal validation sets.

Variables	Total (*N* = 25,101)	Training set (*N* = 17,570)	Validation set (*N* = 7,531)	*P* value
Age (years)	74.00 (70.00, 79.00)	74.00 (70.00, 79.00)	74.00 (69.00, 79.00)	0.981
Sex				0.075
Male	11,592 (46.18)	8,179 (46.55)	3,413 (45.32)	
Female	13,509 (53.82)	9,391 (53.45)	4,118 (54.68)	
Smoking history				0.451
No	18,503 (73.71)	12,927 (73.57)	5,576 (74.04)	
Yes	6,598 (26.29)	4,643 (26.43)	1,955 (25.96)	
Drinking history				0.396
No	20,262 (80.72)	14,158 (80.58)	6,104 (81.05)	
Yes	4,839 (19.28)	3,412 (19.42)	1,427 (18.95)	
Anemia				0.175
No	21,677 (86.36)	15,139 (86.16)	6,538 (86.81)	
Yes	3,424 (13.64)	2,431 (13.84)	993 (13.19)	
COPD				0.873
No	22,498 (89.63)	15,752 (89.65)	6,746 (89.58)	
Yes	2,603 (10.37)	1,818 (10.35)	785 (10.42)	
Cerebral infarction				0.812
No	18,845 (75.08)	13,183 (75.03)	5,662 (75.18)	
Yes	6,256 (24.92)	4,387 (24.97)	1,869 (24.82)	
Osteoporosis				0.963
No	21,369 (85.13)	14,956 (85.12)	6,413 (85.15)	
Yes	3,732 (14.87)	2,614 (14.88)	1,118 (14.85)	
Hypokalemia				0.977
No	21,742 (86.62)	15,220 (86.62)	6,522 (86.60)	
Yes	3,359 (13.38)	2,350 (13.38)	1,009 (13.40)	
CKD				0.137
No	22,035 (87.79)	15,388 (87.58)	6,647 (88.26)	
Yes	3,066 (12.21)	2,182 (12.42)	884 (11.74)	
SBP (mmHg)	137.00 (125.00, 152.00)	137.00 (125.00, 152.00)	137.00 (125.00, 151.00)	0.053
DBP (mmHg)	80.00 (71.00, 87.00)	80.00 (71.00, 87.00)	79.00 (71.00, 87.00)	0.221
Pulse rate (bpm)	80.00 (73.00, 90.00)	80.00 (73.00, 90.00)	80.00 (73.00, 90.00)	0.068
TC (mmol/L)	4.09 (3.35, 4.88)	4.09 (3.35, 4.88)	4.09 (3.35, 4.89)	0.543
TG (mmol/L)	1.36 (0.97, 1.98)	1.36 (0.97, 1.98)	1.36 (0.97, 1.97)	0.955
Cr (µmol/L)	70.00 (56.00, 93.00)	70.00 (56.00, 94.00)	70.00 (56.00, 92.00)	0.063
UA (µmol/L)	324.00 (258.00, 403.00)	325.00 (259.00, 403.00)	322.00 (257.00, 403.00)	0.253
LDL-C (mmol/L)	2.41 (1.75, 3.14)	2.40 (1.74, 3.14)	2.42 (1.76, 3.16)	0.079
HDL-C (mmol/L)	1.03 (0.85, 1.25)	1.03 (0.85, 1.25)	1.04 (0.85, 1.25)	0.472
Hemoglobin (g/L)	126.00 (114.00, 138.00)	127.00 (114.00, 138.00)	126.00 (114.00, 138.00)	0.479
HbA1c (%)	7.40 (6.60, 8.80)	7.40 (6.60, 8.80)	7.40 (6.60, 8.80)	0.921
FG (mmol/L)	7.50 (6.20, 9.80)	7.50 (6.20, 9.80)	7.40 (6.20, 9.80)	0.068
WBC count (×10^9^/L)	6.80 (5.50, 8.70)	6.80 (5.50, 8.70)	6.80 (5.50, 8.60)	0.430
PLT count (×10^9^/L)	184.00 (146.00, 228.00)	185.00 (146.00, 228.00)	184.00 (145.00, 228.00)	0.170
TyG	9.06 (8.61, 9.55)	9.06 (8.61, 9.55)	9.06 (8.60, 9.55)	0.690
TC/HDL-C	3.90 (3.20, 4.77)	3.90 (3.20, 4.77)	3.90 (3.20, 4.77)	0.846
UHR (%)	13.52 (9.75, 18.69)	13.56 (9.76, 18.78)	13.41 (9.72, 18.46)	0.250
AIP	0.49 (0.30, 0.69)	0.49 (0.30, 0.69)	0.48 (0.30, 0.69)	0.554
NHHR	2.90 (2.20, 3.77)	2.90 (2.20, 3.77)	2.90 (2.20, 3.77)	0.846
GHR	7.43 (5.81, 9.66)	7.45 (5.81, 9.66)	7.38 (5.81, 9.63)	0.629
UA/Cr	4.42 (3.42, 5.52)	4.41 (3.41, 5.50)	4.43 (3.44, 5.54)	0.194
SHR	0.82 (0.69, 0.97)	0.82 (0.70, 0.97)	0.82 (0.69, 0.97)	0.025

COPD, chronic obstructive pulmonary disease; CKD, chronic kidney disease; SBP, systolic blood pressure; DBP, diastolic blood pressure; TC, total cholesterol; TG, triglycerides; Cr, creatinine; UA, uric acid; LDL-C, low-density lipoprotein cholesterol; HDL-C, high-density lipoprotein cholesterol; HbA1c, glycated hemoglobin; FG, fasting glucose; WBC, white blood cell; PLT, platelet; TyG, triglyceride glucose; TC/HDL-C, total cholesterol to high-density lipoprotein cholesterol ratio; UHR, uric acid to high-density lipoprotein cholesterol ratio; AIP, atherogenic index of plasma; NHHR, non-high-density lipoprotein cholesterol to high-density lipoprotein cholesterol ratio; GHR, glycated hemoglobin to high-density lipoprotein cholesterol ratio; UA/Cr, uric acid to creatinine ratio; SHR, stress hyperglycemia ratio.

### Variable selection

**Figure 1:**
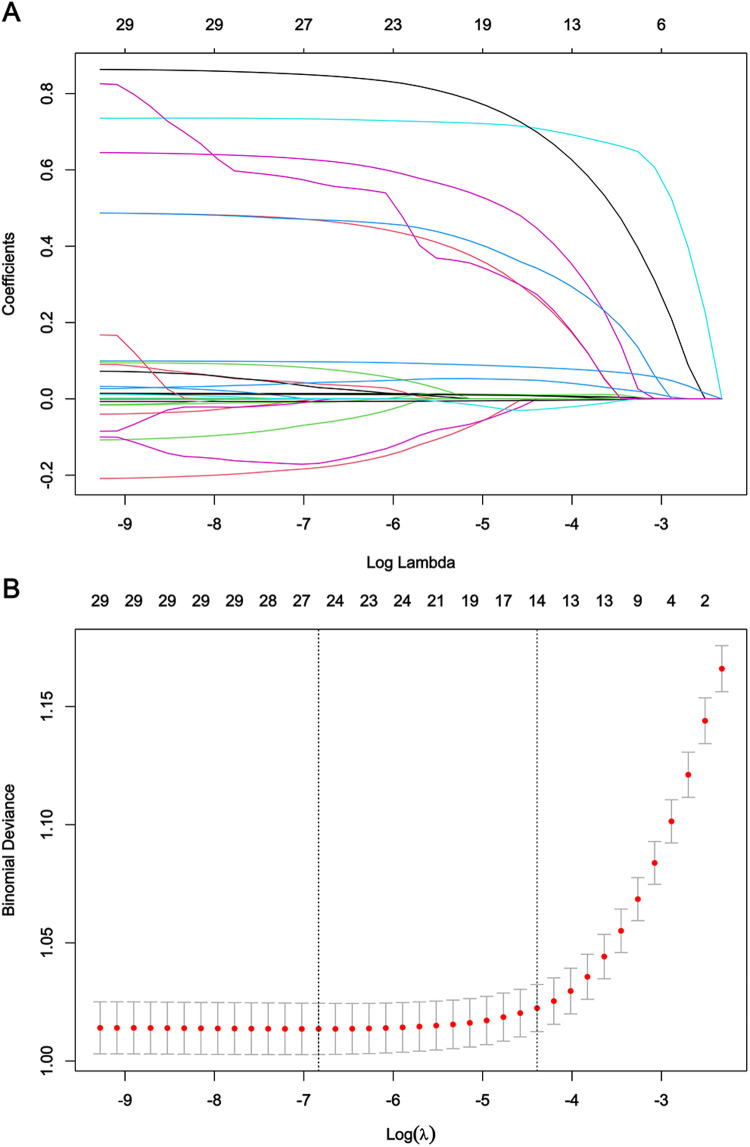
LASSO regression for predictor selection from all candidate variables. **(A)** Regularization paths of coefficients for candidate predictors. Each curve tracks the change in a coefficient as *λ* increases, reflecting the sequential order of predictor entry and the impact of regularization strength on model sparsity. Cross-validation deviance plotted against the log-transformed tuning parameter *λ*. **(B)** Vertical dashed lines indicate the *λ* value minimizing the deviance (left) and the *λ* value one standard error from the minimum (right; lambda.1se = 0.01238537). Variables with non-zero coefficients at lambda.1se were retained as final predictors.

Univariate analysis in the training set revealed that, with the exception of smoking history (*P* = 0.599), drinking history (*P* = 0.386), SBP (*P* = 0.641), TG (*P* = 0.265), and UA (*P* = 0.497), all other variables showed statistically significant differences between the PLOS group and the non-PLOS group (*P* < 0.05) (Table 2). LASSO regression was applied to all candidate variables in the training set. Under the optimal penalty parameter determined by the one-standard-error rule (*λ*_1_se = 0.01238537), a total of 14 variables with non-zero coefficients were retained: age, anemia, COPD, cerebral infarction, osteoporosis, CKD, pulse rate, HDL-C, hemoglobin, FG, WBC count, GHR, UA/Cr, and SHR (Figure 1). To assess multicollinearity among the selected variables, the VIF and its reciprocal (tolerance) for each variable are presented in Supplementary Table S10. All VIF values ranged from 1.022 to 4.089, well below the conventional threshold of 5, indicating no significant multicollinearity among the selected predictors.

**Table 2:** Between-group comparisons of all variables by PLOS status in the training set.

Variables	Total (*N* = 17,570)	PLOS (*N* = 4,741)	Non-PLOS (*N* = 12,829)	*P* value
Age (years)	74.00 (70.00, 79.00)	75.00 (71.00, 81.00)	74.00 (69.00, 79.00)	<0.001
Sex				<0.001
Male	8,179 (46.55)	2,349 (49.55)	5,830 (45.44)	
Female	9,391 (53.45)	2,392 (50.45)	6,999 (54.56)	
Smoking history				0.599
No	12,927 (73.57)	3,474 (73.28)	9,453 (73.68)	
Yes	4,643 (26.43)	1,267 (26.72)	3,376 (26.32)	
Drinking history				0.386
No	14,158 (80.58)	3,841 (81.02)	10,317 (80.42)	
Yes	3,412 (19.42)	900 (18.98)	2,512 (19.58)	
Anemia				<0.001
No	15,139 (86.16)	3,491 (73.63)	11,648 (90.79)	
Yes	2,431 (13.84)	1,250 (26.37)	1,181 (9.21)	
COPD				<0.001
No	15,752 (89.65)	4,026 (84.92)	11,726 (91.40)	
Yes	1,818 (10.35)	715 (15.08)	1,103 (8.60)	
Cerebral infarction				<0.001
No	13,183 (75.03)	2,971 (62.67)	10,212 (79.60)	
Yes	4,387 (24.97)	1,770 (37.33)	2,617 (20.40)	
Osteoporosis				<0.001
No	14,956 (85.12)	3,845 (81.10)	11,111 (86.61)	
Yes	2,614 (14.88)	896 (18.90)	1,718 (13.39)	
Hypokalemia				0.006
No	15,220 (86.62)	4,051 (85.45)	11,169 (87.06)	
Yes	2,350 (13.38)	690 (14.55)	1,660 (12.94)	
CKD				<0.001
No	15,388 (87.58)	3,759 (79.29)	11,629 (90.65)	
Yes	2,182 (12.42)	982 (20.71)	1,200 (9.35)	
SBP (mmHg)	137.00 (125.00, 152.00)	137.00 (125.00, 153.00)	138.00 (126.00, 152.00)	0.641
DBP (mmHg)	80.00 (71.00, 87.00)	79.00 (70.00, 87.00)	80.00 (72.00, 87.00)	<0.001
Pulse rate (bpm)	80.00 (73.00, 90.00)	85.00 (75.00, 96.00)	80.00 (72.00, 89.00)	<0.001
TC (mmol/L)	4.09 (3.35, 4.88)	3.94 (3.18, 4.75)	4.14 (3.41, 4.92)	<0.001
TG (mmol/L)	1.36 (0.97, 1.98)	1.34 (0.98, 1.95)	1.37 (0.97, 2.00)	0.265
Cr (µmol/L)	70.00 (56.00, 94.00)	76.00 (59.00, 113.00)	69.00 (56.00, 89.00)	<0.001
UA (µmol/L)	325.00 (259.00, 403.00)	328.00 (252.00, 415.00)	324.00 (261.00, 399.00)	0.497
LDL-C (mmol/L)	2.40 (1.74, 3.14)	2.29 (1.61, 3.07)	2.44 (1.79, 3.16)	<0.001
HDL-C (mmol/L)	1.03 (0.85, 1.25)	0.97 (0.78, 1.18)	1.05 (0.88, 1.27)	<0.001
Hemoglobin (g/L)	127.00 (114.00, 138.00)	121.00 (105.00, 135.00)	128.00 (117.00, 139.00)	<0.001
HbA1c (%)	7.40 (6.60, 8.80)	7.50 (6.60, 9.20)	7.40 (6.60, 8.60)	<0.001
FG (mmol/L)	7.50 (6.20, 9.80)	8.20 (6.50, 11.10)	7.30 (6.10, 9.40)	<0.001
WBC count (×10^9^/L)	6.80 (5.50, 8.70)	7.70 (6.00, 10.30)	6.60 (5.30, 8.20)	<0.001
PLT count (×10^9^/L)	185.00 (146.00, 228.00)	188.00 (144.00, 237.00)	184.00 (147.00, 225.00)	0.001
TyG	9.06 (8.61, 9.55)	9.12 (8.68, 9.64)	9.03 (8.59, 9.52)	<0.001
TC/HDL-C	3.90 (3.20, 4.77)	4.00 (3.28, 4.96)	3.87 (3.17, 4.70)	<0.001
UHR (%)	13.56 (9.76, 18.78)	14.52 (10.18, 20.60)	13.29 (9.64, 18.16)	<0.001
AIP	0.49 (0.30, 0.69)	0.51 (0.33, 0.72)	0.48 (0.29, 0.68)	<0.001
NHHR	2.90 (2.20, 3.77)	3.00 (2.28, 3.96)	2.87 (2.17, 3.70)	<0.001
GHR	7.45 (5.81, 9.66)	8.13 (6.26, 10.88)	7.21 (5.69, 9.25)	<0.001
UA/Cr	4.41 (3.41, 5.50)	4.02 (2.90, 5.18)	4.53 (3.57, 5.61)	<0.001
SHR	0.82 (0.70, 0.97)	0.86 (0.72, 1.05)	0.81 (0.69, 0.95)	<0.001

COPD, chronic obstructive pulmonary disease; CKD, chronic kidney disease; SBP, systolic blood pressure; DBP, diastolic blood pressure; TC, total cholesterol; TG, triglycerides; Cr, creatinine; UA, uric acid; LDL-C, low-density lipoprotein cholesterol; HDL-C, high-density lipoprotein cholesterol; HbA1c, glycated hemoglobin; FG, fasting glucose; WBC, white blood cell; PLT, platelet; TyG, triglyceride glucose; TC/HDL-C, total cholesterol to high-density lipoprotein cholesterol ratio; UHR, uric acid to high-density lipoprotein cholesterol ratio; AIP, atherogenic index of plasma; NHHR, non-high-density lipoprotein cholesterol to high-density lipoprotein cholesterol ratio; GHR, glycated hemoglobin to high-density lipoprotein cholesterol ratio; UA/Cr, uric acid to creatinine ratio; SHR, stress hyperglycemia ratio.

Table 2 presents univariate comparisons between PLOS and non-PLOS groups for descriptive purposes only; these comparisons were not used as a prescreening step for subsequent LASSO regression.

### Nonlinear associations of continuous variables

**Figure 2:**
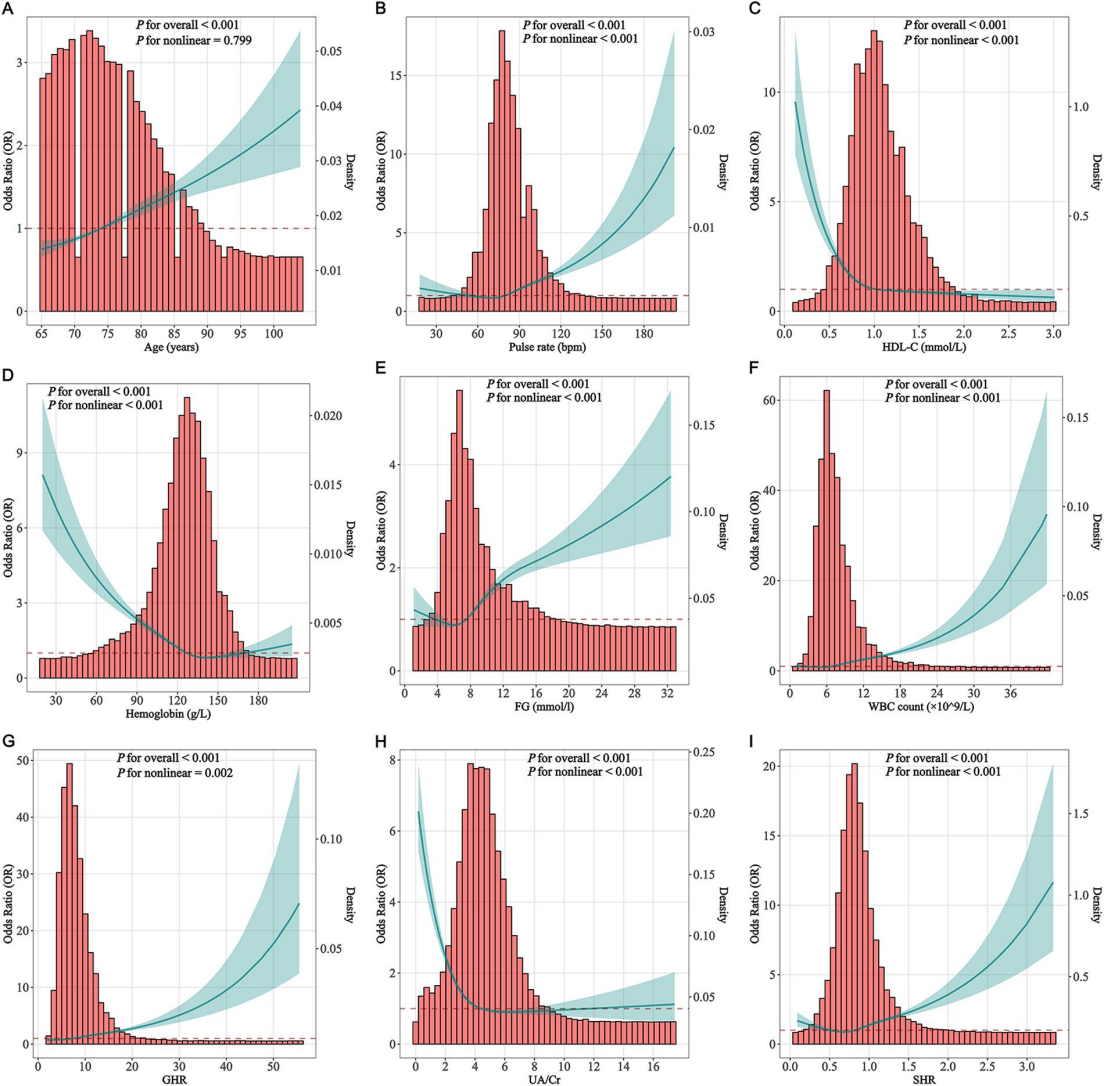
Restricted cubic spline (RCS) analysis of dose-response relationships between LASSO-selected continuous variables and PLOS risk. **(A)** Age; **(B)** pulse rate; **(C)** HDL-C; **(D)** hemoglobin; **(E)** FG; **(F)** WBC count; **(G)** GHR; **(H)** UA/Cr; **(I)** SHR.

RCS regression was performed to characterize the dose-response relationships between the nine continuous predictors selected by LASSO and the risk of PLOS in older patients with T2DM complicated by CVD (Figure 2). All nine variables demonstrated statistically significant overall associations with PLOS risk (all *P*-overall < 0.001). With respect to nonlinearity, age exhibited an approximately linear relationship with PLOS risk (*P*-nonlinear = 0.799). By contrast, pulse rate, HDL-C, hemoglobin, FG, WBC count, UA/Cr, and SHR each showed pronounced nonlinear patterns (all *P*-nonlinear < 0.001). Furthermore, GHR also displayed a significant departure from linearity (*P*-nonlinear = 0.002). Collectively, these results indicate that while all nine continuous variables are significantly associated with PLOS risk, the shapes of their associations vary considerably, with age being the only variable following a near-linear dose-response relationship. However, as these associations were derived from a retrospective observational dataset, they should be interpreted as predictive associations rather than causal intervention targets. Prospective studies are needed to confirm whether modifying these variables would actually influence length of stay.

### ML model development and performance comparison

**Figure 3:**
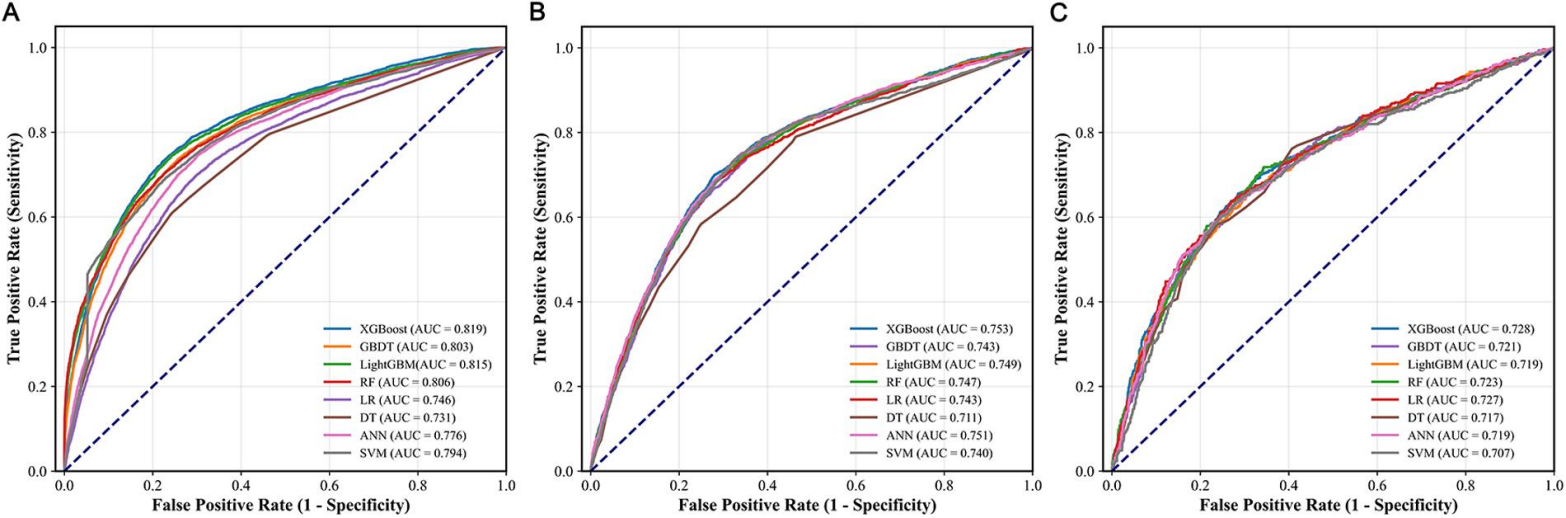
Receiver operating characteristic (ROC) curves and area under the curve (AUC) for eight ML models. **(A)** Training set; **(B)** internal validation set; **(C)** external validation set.

Among the eight predictive models developed in this study, XGBoost achieved the highest AUC in the training set (0.819, 95% CI: 0.811–0.826) and demonstrated competitive performance in both the internal validation set (AUC = 0.753, 95% CI: 0.740–0.766) and the external validation set (AUC = 0.728, 95% CI: 0.700–0.751) (Figure 3). However, the performance gap between XGBoost and simpler models narrowed substantially in validation: LR achieved comparable AUCs (internal: 0.743, 95% CI: 0.731–0.755; external: 0.728, 95% CI: 0.705–0.754) with considerably less overfitting (training-to-validation AUC drop: 0.003 vs. 0.066 for XGBoost). Pairwise DeLong tests revealed that XGBoost did not achieve statistically significantly higher AUC than LR in the external validation set (*P* = 0.369). Similarly, no statistically significant differences were observed between XGBoost and ANN in the external validation set (*P* = 0.236). These results indicate that the apparent advantage of XGBoost over simpler models was not statistically robust in held-out data. Regarding PR-AUC, the XGBoost model achieved 0.685 (95% CI: 0.672–0.698) in the training set, 0.502 (95% CI: 0.480–0.526) in the internal validation set, and 0.424 (95% CI: 0.383–0.473) in the external validation set. For LR, the corresponding PR-AUC values were 0.511 (95% CI: 0.496–0.526), 0.496 (95% CI: 0.473–0.521), and 0.412 (95% CI: 0.373–0.458), respectively. The PR-AUC results were consistent with the ROC-AUC findings, showing modest differences that did not favor XGBoost substantially in validation. The full PR-AUC results for all eight models across all three datasets are reported in Supplementary Table S11. Calibration metrics for XGBoost and LR—the two models with the closest performance in validation—are summarized in Table 5 for direct comparison, and the complete calibration results (Brier score, calibration slope, and calibration intercept) for all eight models across all three datasets are provided in Supplementary Table S11.

Other models, including ANN (internal AUC = 0.752, 95% CI: 0.738–0.764) and SVM (internal AUC = 0.740, 95% CI: 0.727–0.754), also showed competitive validation performance. Regarding classification performance in the training set, the XGBoost model yielded a sensitivity of 0.732 (95% CI: 0.719–0.744), a specificity of 0.777 (95% CI: 0.770–0.784), a Youden index of 0.508, and an F1 score of 0.627 (95% CI: 0.616–0.637) (Table 3). In the internal validation set, the corresponding values were 0.662 (95% CI: 0.641–0.684), 0.743 (95% CI: 0.732–0.755), 0.405, and 0.551 (95% CI: 0.533–0.568), respectively; in the external validation set, the values were 0.638 (95% CI: 0.596–0.677), 0.722 (95% CI: 0.703–0.741), 0.360, and 0.466 (95% CI: 0.431–0.496), respectively (Table 4; Supplementary Table S12). The training-derived Youden-optimized threshold for the XGBoost model was 0.286; the corresponding thresholds for all eight models are listed in Supplementary Table S11.

**Table 3:** Predictive performance of eight ML models for PLOS risk in the training set.

Model	AUC (95% CI)	Sensitivity (95% CI)	Specificity (95% CI)	F1 score (95% CI)	Youden index
XGBoost	0.819 (0.811–0.826)	0.732 (0.719–0.744)	0.777 (0.770–0.784)	0.627 (0.616–0.637)	0.508
GBDT	0.803 (0.795–0.811)	0.747 (0.734–0.759)	0.733 (0.725–0.740)	0.604 (0.594–0.615)	0.479
LightGBM	0.815 (0.808–0.822)	0.734 (0.722–0.747)	0.769 (0.762–0.776)	0.623 (0.612–0.632)	0.503
RF	0.806 (0.799–0.814)	0.705 (0.692–0.718)	0.773 (0.765–0.780)	0.608 (0.597–0.619)	0.478
LR	0.746 (0.738–0.754)	0.695 (0.682–0.708)	0.705 (0.698–0.713)	0.558 (0.548–0.568)	0.400
DT	0.731 (0.723–0.739)	0.610 (0.597–0.623)	0.756 (0.749–0.764)	0.537 (0.526–0.548)	0.366
ANN	0.778 (0.769–0.786)	0.751 (0.738–0.764)	0.694 (0.686–0.702)	0.583 (0.572–0.594)	0.445
SVM	0.794 (0.786–0.801)	0.694 (0.681–0.707)	0.769 (0.762–0.777)	0.599 (0.588–0.609)	0.463

AUC, area under the ROC curve; CI, confidence interval; XGBoost, extreme Gradient Boosting; GBDT, gradient boosting decision tree; LightGBM, light gradient boosting machine; RF, random forest; LR, logistic regression; DT, decision tree; ANN, artificial neural network; SVM, support vector machine. All sensitivity, specificity, F1, and Youden index values were calculated using model-specific thresholds derived from the training set by maximizing the Youden index. These thresholds are reported in Supplementary Table S11.

**Table 4:** Predictive performance of eight ML models for PLOS risk in the internal validation set.

Model	AUC (95% CI)	Sensitivity (95% CI)	Specificity (95% CI)	F1 score (95% CI)	Youden index
XGBoost	0.753 (0.740–0.766)	0.662 (0.641–0.684)	0.743 (0.732–0.755)	0.551 (0.533–0.568)	0.405
GBDT	0.743 (0.730–0.755)	0.674 (0.655–0.696)	0.708 (0.696–0.720)	0.536 (0.519–0.553)	0.382
LightGBM	0.749 (0.736–0.761)	0.642 (0.622–0.664)	0.747 (0.736–0.758)	0.542 (0.524–0.559)	0.389
RF	0.748 (0.734–0.759)	0.642 (0.620–0.661)	0.750 (0.739–0.760)	0.543 (0.524–0.560)	0.392
LR	0.743 (0.731–0.755)	0.686 (0.667–0.707)	0.714 (0.702–0.726)	0.547 (0.531–0.563)	0.400
DT	0.711 (0.697–0.723)	0.584 (0.561–0.604)	0.750 (0.740–0.762)	0.507 (0.490–0.525)	0.334
ANN	0.752 (0.738–0.764)	0.722 (0.703–0.741)	0.683 (0.671–0.695)	0.548 (0.532–0.564)	0.404
SVM	0.740 (0.727–0.754)	0.637 (0.617–0.661)	0.752 (0.740–0.762)	0.542 (0.525–0.560)	0.389

AUC, area under the ROC curve; CI, confidence interval; XGBoost, extreme Gradient Boosting; GBDT, gradient boosting decision tree; LightGBM, light gradient boosting machine; RF, random forest; LR, logistic regression; DT, decision tree; ANN, artificial neural network; SVM, support vector machine. All sensitivity, specificity, F1, and Youden index values were calculated using model-specific thresholds derived from the training set by maximizing the Youden index. These thresholds are reported in Supplementary Table S11.

**Figure 4:**
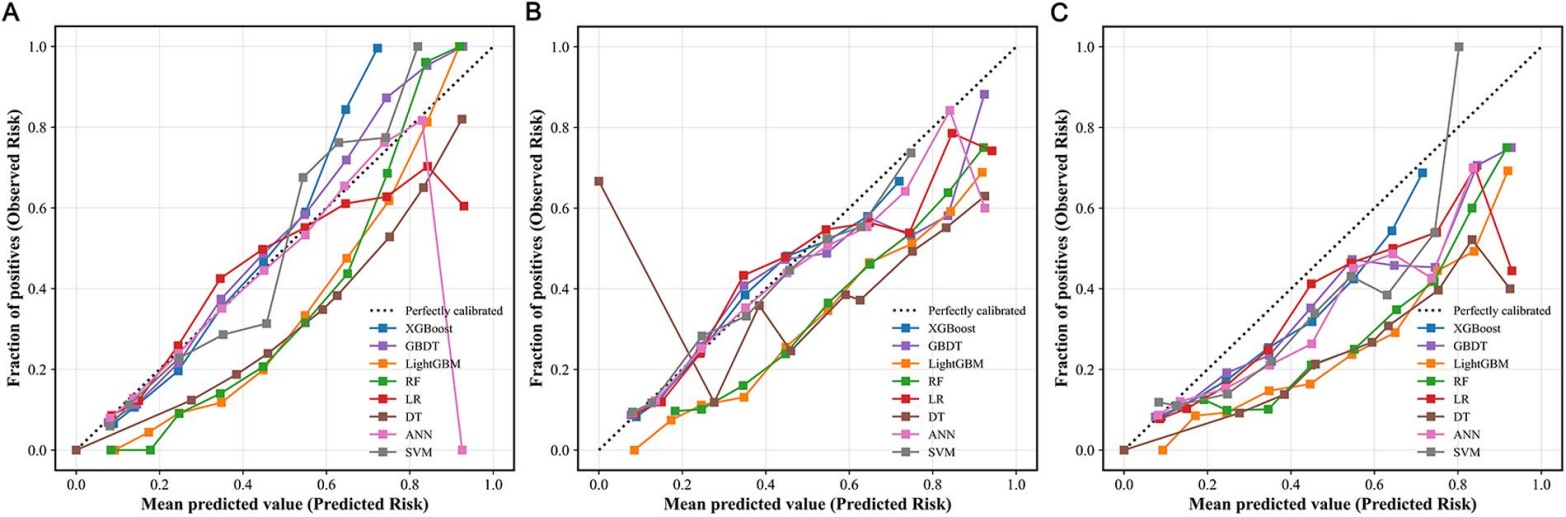
Calibration curves comparing predicted vs. observed PLOS risk across eight ML models. **(A)** Training set; **(B)** internal validation set; **(C)** external validation set.

**Figure 5:**
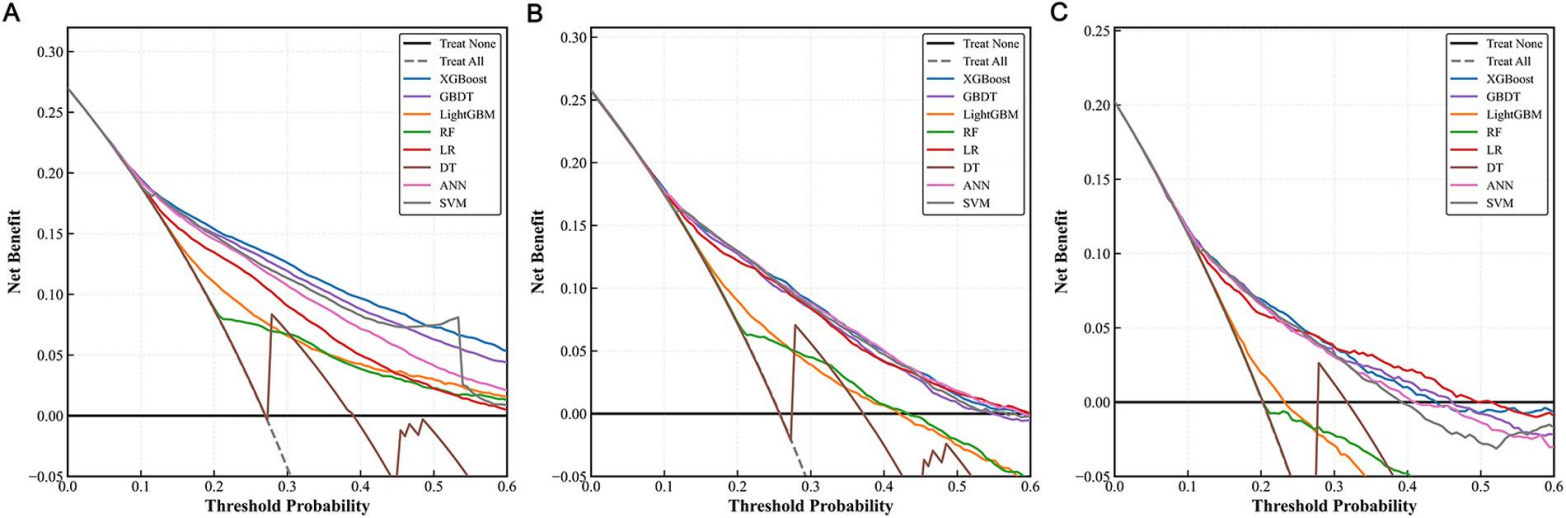
Decision curve analysis (DCA) comparing net benefit across eight ML models across a range of threshold probabilities. **(A)** Training set; **(B)** internal validation set; **(C)** external validation set.

The calibration curves showed generally acceptable agreement between predicted and observed probabilities in the training and internal validation sets, but revealed systematic deviations in the external validation set (Figure 4). Numerical calibration metrics for the XGBoost model further quantified these observations, with 95% CIs estimated via bootstrap. In the training set, the Brier score was 0.143 (95% CI: 0.140–0.146), the calibration slope was 1.393 (95% CI: 1.349–1.438), and the calibration intercept was 0.310 (95% CI: 0.260–0.360) (Supplementary Table S11). In the internal validation set, the corresponding values were 0.161 (95% CI: 0.156–0.165), 1.013 (95% CI: 0.955–1.077), and −0.039 (95% CI: −0.115 to 0.037), respectively, indicating satisfactory calibration in this temporally matched cohort. In the external validation set, however, the Brier score was 0.147 (95% CI: 0.138–0.154), the calibration slope was 0.889 (95% CI: 0.790–1.003), and the calibration intercept was −0.484 (95% CI: −0.606 to −0.335), suggesting systematic miscalibration—specifically, the model tended to overestimate risk at lower predicted probabilities and underestimate risk at higher predicted probabilities in the temporally distinct population. The calibration performance of LR was comparable to that of XGBoost in the internal validation set (intercept: −0.074, 95% CI: −0.159 to 0.014; slope: 0.977, 95% CI: 0.908–1.048) and showed similarly systematic deviations in the external set (intercept: −0.513, 95% CI: −0.665 to −0.373; slope: 0.916, 95% CI: 0.795–1.048) (Table 5), indicating that the calibration drift in the external cohort affected both models. DCA demonstrated that the XGBoost model provided positive net benefit across a range of clinically relevant threshold probabilities (0%–60%), indicating that interventions guided by the model would benefit patients without causing net harm (Figure 5). The net benefit was comparable to that of LR across most threshold ranges, consistent with the similar AUCs. Based on these comprehensive evaluations, the XGBoost model was selected as the final predictive model for this study with the understanding that local recalibration would be necessary before deployment in new clinical settings.

**Table 5:** Performance and calibration metrics for the XGBoost and LR models across three datasets.

Model	Dataset	PR-AUC (95% CI)	Brier score (95% CI)	Calibration intercept (95% CI)	Calibration slope (95% CI)	Threshold used
XGBoost	Training	0.685 (0.672–0.698)	0.143 (0.140–0.146)	0.310 (0.260–0.360)	1.393 (1.349–1.438)	0.286
XGBoost	Internal validation	0.502 (0.480–0.526)	0.161 (0.156–0.165)	−0.039 (−0.115 to 0.037)	1.013 (0.955–1.077)	0.286
XGBoost	External validation	0.424 (0.383–0.473)	0.147 (0.138–0.154)	−0.484 (−0.606 to −0.335)	0.889 (0.790–1.003)	0.286
LR	Training	0.511 (0.496–0.526)	0.168 (0.165–0.171)	0.005 (−0.049 to 0.063)	1.006 (0.960–1.050)	0.250
LR	Internal validation	0.496 (0.473–0.521)	0.165 (0.160–0.170)	−0.074 (−0.159 to 0.014)	0.977 (0.908–1.048)	0.250
LR	External validation	0.412 (0.373–0.458)	0.147 (0.140–0.155)	−0.513 (−0.665 to −0.373)	0.916 (0.795–1.048)	0.250

PR, precision-recall; AUC, area under the ROC curve; CI, confidence interval; XGBoost, extreme Gradient Boosting; LR, logistic regression.

### Sensitivity analysis of PLOS definition

To assess whether the choice of PLOS threshold materially influenced model performance and whether the model remained robust in unseen patients, we performed sensitivity analyses using the XGBoost model with two alternative PLOS definitions: the 90th percentile of the training set distribution (≥18 days) and a fixed cutoff of 14 days. For each alternative definition, the model was retrained in the training set using the same hyperparameter tuning procedure and evaluated in both validation sets. Under the 90th percentile definition (≥18 days), the XGBoost model achieved an AUC of 0.806 (95% CI: 0.796–0.818) in the training set, 0.741 (95% CI: 0.725–0.756) in the internal validation set, and 0.719 (95% CI: 0.691–0.745) in the external validation set. Under the fixed 14-day cutoff, the corresponding AUCs were 0.809 (95% CI: 0.801–0.818), 0.748 (95% CI: 0.734–0.762), and 0.725 (95% CI: 0.698–0.751), respectively. These values were comparable to those obtained with our primary 75th percentile definition as reported above. These findings indicate that the XGBoost model maintained consistent discriminative performance across different PLOS thresholds not only in the training set but also in unseen validation cohorts, supporting the robustness of our primary definition rather than its superiority.

### Robustness of the imputation strategy

To assess whether the choice of imputed dataset affected model performance, we fitted the XGBoost model on each of the five imputed datasets and compared the resulting performance metrics. The AUCs across the five datasets ranged from 0.817 to 0.820 (mean: 0.819, standard deviation: 0.001), with similarly minimal variability observed for sensitivity, specificity, F1 score, and Brier score (Supplementary Table S13). These results confirmed that using the first imputed dataset did not materially affect model performance.

### Model application

**Figure 6:**
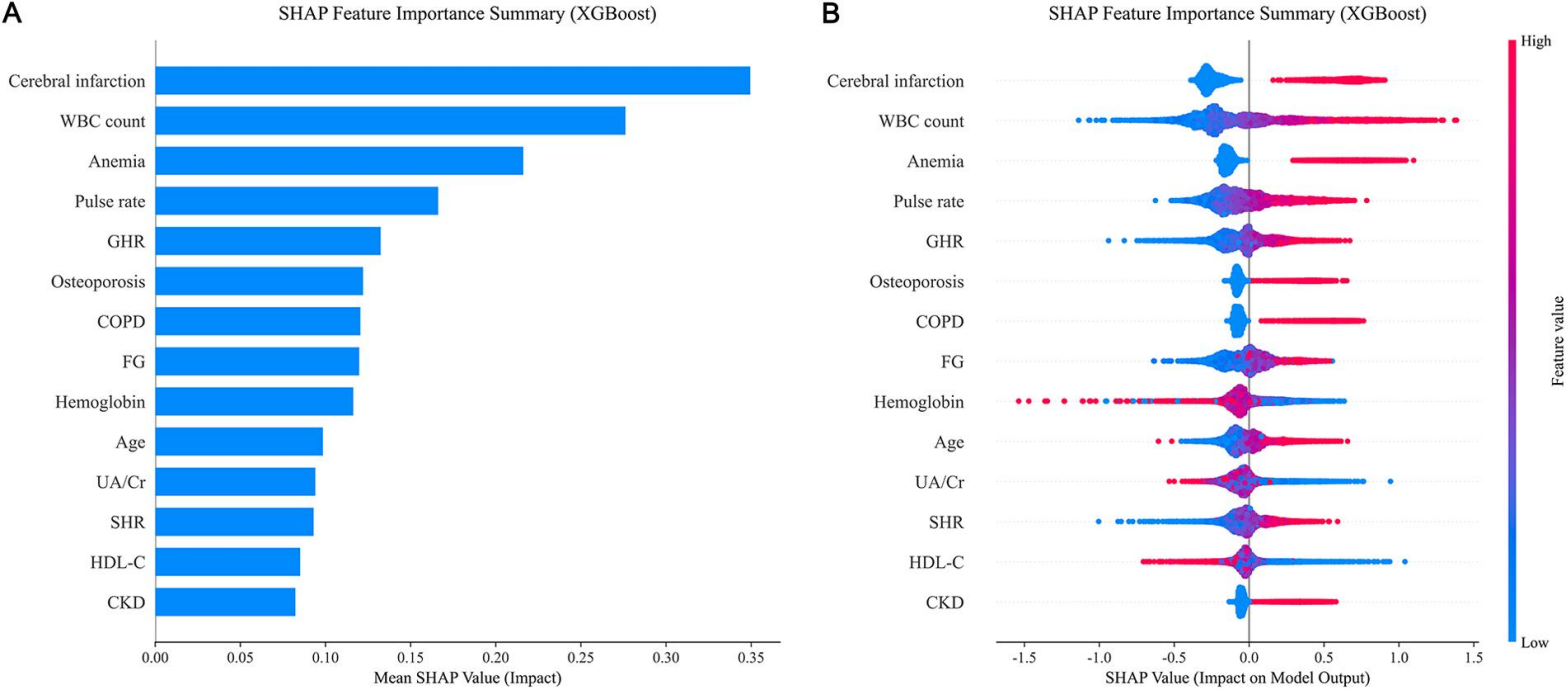
SHAP (shapley additive explanations) analysis for interpreting the XGBoost model. **(A)** Bar chart of SHAP-based feature importance. **(B)** Summary dot plot: red and blue denote high and low feature values, respectively; SHAP values on the left side of the vertical axis reduce the predicted PLOS risk, whereas those on the right side increase it.

SHAP analysis was performed to evaluate feature importance and the direction of their contributions within the final XGBoost model. According to the SHAP results, the six most influential predictors—ranked by their overall impact on model output—were cerebral infarction, WBC count, anemia, pulse rate, GHR, and osteoporosis (Figure 6A). Regarding the direction of effect, a total of eleven variables, including cerebral infarction, WBC count, and anemia, were found to substantially increase the likelihood of PLOS, as indicated by the red bars in Figure 6B. In contrast, hemoglobin, HDL-C, and UA/Cr were associated with a reduced risk of PLOS, as shown by the blue bars in the same figure. Figure 6B further illustrates both the magnitude and the direction of each feature's contribution to PLOS risk prediction.

## Discussion

Leveraging real-world clinical data, this study systematically developed and compared eight ML models to predict PLOS among older patients with T2DM complicated by CVD. Among all models evaluated, XGBoost demonstrated the highest training AUC and competitive validation performance, with an internal validation AUC of 0.753 and an external validation AUC of 0.728. It also exhibited favorable calibration in internal validation and demonstrated a favorable net clinical benefit across clinically relevant threshold probabilities. SHAP analysis further identified a history of cerebral infarction, WBC count, anemia, pulse rate, GHR, and osteoporosis as the six most influential predictors driving model outputs. It is important to note, however, that the performance advantage of XGBoost over simpler models such as logistic regression was modest in validation, and we therefore present this model as one of several viable approaches rather than a definitively superior tool. Collectively, these findings enrich the existing evidence base for predicting PLOS in this multimorbid population and suggest a quantifiable decision-making framework for early risk identification in clinical practice. Nevertheless, these results should be interpreted as proof-of-concept evidence, and prospective implementation studies are needed to evaluate the model's impact on clinical decision-making and patient outcomes in real-world settings.

This finding aligns with previous research: Jaotombo et al. compared five ML models for predicting PLOS using a French medico-administrative database of 73,182 hospitalizations and reported that the gradient boosting classifier achieved the highest AUC of 0.810, significantly outperforming LR and other models (25). Among cardiac patient populations, Daghistani et al. similarly confirmed that XGBoost offers higher accuracy and robustness for predicting hospital stay duration (26). Furthermore, Deschepper et al. applied XGBoost to predict binary PLOS in adult acute care patients, reporting an AUC of 0.94, further supporting the strong discriminative capacity of this model in heterogeneous hospital populations (27). Notably, although Tan and colleagues developed a nomogram-based PLOS prediction model for older T2DM patients, its reported AUC of 0.803 was modestly lower than the performance of our XGBoost model, suggesting that ML approaches may provide incremental predictive value in complex chronic disease populations (21). Moreover, a recent multi-database external validation study by Allyn J et al. demonstrated that single-source training consistently overestimates model performance, with mean absolute percentage error increasing by up to 51.8% and AUC dropping by up to 13.8% when applied to external cohorts (28). This finding directly echoes the observed decline in our internal validation set and further underscores the value of our time-based external validation, which confirmed that the model maintained consistent discriminative performance (AUC = 0.728) in a temporally independent patient cohort.

The comparable performance between XGBoost and logistic regression in our validation sets warrants explicit discussion. In our study, XGBoost achieved the highest training AUC (0.819) but showed a substantial drop in validation (internal: 0.753; external: 0.728; drop: 0.066). In contrast, LR demonstrated stable performance across training and validation (AUC: 0.746 vs. 0.743 vs. 0.728; drop: 0.003) and better calibration (validation calibration intercept: −0.074 vs. −0.041 for XGBoost). Formal pairwise DeLong tests confirmed that the AUC differences between XGBoost and LR was not statistically significant in the external validation set (*P* = 0.369), and PR-AUC values were likewise comparable (XGBoost vs. LR: 0.502 vs. 0.496 in internal validation; 0.424 vs. 0.412 in external validation). This pattern suggests that XGBoost captures more complex patterns in the training data, but these patterns do not fully generalize to new populations. Given these findings, we acknowledge that LR represents a parsimonious and equally effective alternative that may be preferable in settings where simplicity and interpretability are prioritized. In this study, we selected XGBoost as the primary model for the following reasons: (1) it maintained the highest training AUC and competitive validation AUCs; (2) it offers the capability to capture complex nonlinear relationships and interactions among predictors, as demonstrated by the RCS analyses, which showed that most continuous variables exhibited nonlinear associations with PLOS risk—patterns that linear models such as LR cannot fully capture; (3) it provides SHAP-based interpretability to identify key predictive associations and visualize nonlinear dose-response patterns; and (4) it has been widely adopted in prior clinical prediction studies, facilitating comparison with existing literature. We emphasize, however, that the performance advantage of XGBoost over LR was not statistically significant in validation, and the model should therefore be viewed as one of several viable approaches rather than a definitively superior tool. For institutions seeking a simpler, more interpretable model with comparable validation performance, LR represents a well-justified alternative. Future research with larger and more diverse cohorts is needed to determine whether the added complexity of XGBoost translates into meaningful clinical benefit.

The predictive accuracy of ML models often comes at the cost of interpretability, which constitutes a major barrier to clinical translation (29). In this study, we employed SHAP analysis to provide *post-hoc* explanations for the XGBoost model, quantifying the marginal contribution of each feature to the prediction outcome. The results identified cerebral infarction, WBC count, anemia, pulse rate, GHR, and osteoporosis as the six most influential predictors. This finding aligns with SHAP-based applications in cardiovascular risk prediction: a study on heart failure readmission similarly identified inflammatory markers (WBC count) and comorbidity burden (anemia) as key drivers (30). Of note, Oka and Takefuji recently raised concerns regarding the reliability of ML-derived feature importance, noting that SHAP values may reflect model bias rather than true causal relationships, particularly in high-dimensional data with collinearity (31). To mitigate this concern, we pre-screened variables using LASSO regression to address multicollinearity (all VIFs < 5), and we presented directional contributions alongside importance rankings to enhance the robustness of the explanations. The Interpretable Ensemble Learning Framework further demonstrated that combining SHAP with Local Interpretable Model-agnostic Explanations (LIME) can improve the cross-sample stability of feature attributions (32).

The six core predictors identified in this study each possess clear clinical pathophysiological plausibility, and RCS analysis further revealed nonlinear dose-response relationships with PLOS risk. A history of cerebral infarction exhibited a step-like effect, with risk increasing sharply after its occurrence (33). WBC count demonstrated a J-shaped relationship, with risk accelerating substantially beyond 10 × 10^9^/L. Hemoglobin concentration showed an inverted L-shaped relationship, with risk rising steeply below 110 g/L. Although multiple prospective studies have confirmed that preoperative anemia is an independent risk factor for PLOS (34, 35), our findings reflect predictive associations within our model rather than causal effects; these patterns should not be interpreted as evidence that correcting hemoglobin levels to specific targets would necessarily reduce PLOS risk. The slope of pulse rate steepened notably above 90 beats per minute, indicating tachycardia as a critical signal for poor prognosis (36). The risk of PLOS increased slowly at lower GHR levels, but the ascending slope steepened progressively as GHR rose, indicating a nonlinear accelerating risk pattern associated with combined hyperglycemia and low HDL-C (37). Osteoporosis was associated with a significantly increased risk of PLOS, likely mediated by increased fracture susceptibility, reduced mobility, and prolonged bed rest during hospitalization (38). These findings provide refined, individualized thresholds for clinical intervention. However, these thresholds should be interpreted as descriptive patterns observed in our predictive model, not as evidence-based treatment targets. Clinical decision-making should continue to rely on established guidelines and prospective evidence.

Regarding model calibration, we observed that the calibration intercept in the training set was 0.310 (95% CI: 0.260–0.360), indicating some miscalibration during training (systematic underestimation of risk). This improved substantially in the internal validation set (intercept = −0.039, 95% CI: −0.115 to 0.037; slope = 1.013, 95% CI: 0.955–1.077), demonstrating satisfactory calibration in this temporally matched cohort. In the external validation set, however, the calibration intercept was −0.484 (95% CI: −0.606 to −0.335) and the slope was 0.889 (95% CI: 0.790–1.003). These external calibration metrics indicate systematic miscalibration: the negative intercept suggests overestimation of risk at lower predicted probabilities, while the slope <1.0 suggests underestimation at higher predicted probabilities. Notably, LR exhibited similar calibration drift in the external set (intercept: −0.513, 95% CI: −0.665 to −0.373; slope: 0.916, 95% CI: 0.795–1.048), suggesting that this temporal shift was not unique to XGBoost but reflected broader changes in the patient population or clinical practices over time. These findings have important implications for clinical translation. The statistically significant miscalibration observed in the external cohort underscores that local recalibration is essential before deploying the model in new settings. We recommend that institutions planning to adopt this model perform intercept adjustment or slope re-estimation using their own data, and incorporate ongoing calibration monitoring as part of the model maintenance plan.

While these results are encouraging, it is important to recognize that predictive model performance in retrospective studies does not guarantee clinical utility. The ultimate value of our model can only be established through prospective implementation studies that assess whether providing PLOS risk predictions to clinicians actually changes clinical management and improves patient outcomes or resource utilization. Until such studies are conducted, the clinical implications of our findings should be considered preliminary.

Several limitations should be acknowledged in this study. First, despite the use of multiple imputation to address missing data, the retrospective design cannot fully eliminate selection bias or the influence of unmeasured confounders. Specifically, we used the first of five imputed datasets for model development after confirming minimal between-imputation variability. While we assessed the stability of model performance across datasets and found no meaningful differences, we acknowledge that this simplified approach does not formally propagate imputation uncertainty. This limitation should be considered when interpreting the results, although the low missing rates and minimal imputation variability suggest that the impact on our conclusions is likely negligible. Second, while we have performed time-based external validation using a temporally distinct cohort from the same institution, all data were still derived from a single medical center. External validation in geographically and institutionally diverse healthcare settings would further strengthen the generalizability of our model. Third, the decline in XGBoost performance from training to validation (AUC drop of 0.066) indicates some degree of overfitting, which is not uncommon when applying complex ML models to clinical data. We attempted to mitigate this through LASSO-based feature selection, 5-fold cross-validation during hyperparameter tuning, and early stopping for XGBoost and LightGBM. However, the observed overfitting suggests that simpler models such as LR may be equally or more appropriate in some settings, and we encourage users of our model to consider local recalibration before deployment. Fourth, PLOS was defined as exceeding the 75th percentile of the training set (≥12 days). This threshold may not be uniformly applicable across different healthcare settings or discharge practices, as length of stay is highly dependent on local bed availability, insurance systems, and clinical workflows. We acknowledge that this institution-specific threshold may limit the generalizability of our findings to settings with substantially different discharge practices. However, our sensitivity analyses using alternative thresholds (90th percentile and a fixed cutoff of 14 days), with model retraining and evaluation in both validation cohorts, demonstrated consistent performance across all three PLOS definitions: internal validation AUCs ranged from 0.741 to 0.753, and external validation AUCs ranged from 0.719 to 0.728. These findings suggest that the predictive ability of the model is robust to the choice of PLOS definition and generalizes to unseen patients across different outcome definitions. We acknowledge that we did not perform a continuous or ordinal analysis of length of stay, as our primary objective was to develop a binary prediction tool for clinical decision-making (i.e., identifying patients at risk of prolonged hospitalization). However, we recognize that examining length of stay as a continuous or ordinal outcome could provide additional insights, and we have noted this as a direction for future research. Nonetheless, external validation in diverse healthcare systems with different baseline length-of-stay distributions is warranted to further assess the transportability of our model. Fifth, although we have ensured that all predictors were measured at admission or within 24 h, the specific timing of laboratory measurements may vary in clinical practice, and delays in test results could affect the real-time applicability of the model. Moreover, certain composite indices rely on laboratory data that may not be immediately available at the exact moment of admission; however, as these results are typically obtained within hours, they remain suitable for early risk stratification. Sixth, we excluded patients who died during hospitalization. While this exclusion was clinically justified—as predicting PLOS in terminally ill patients has limited clinical utility and mortality represents a competing outcome—we acknowledge that this may limit the generalizability of our model to the most severely ill patients. Additionally, although we applied XGBoost's built-in scale_pos_weight to account for class imbalance, other class-balancing procedures such as oversampling or synthetic data generation were not used. Future studies may consider treating death as a competing risk or developing separate models for patients at different severity levels. Furthermore, key social determinants of health, including socioeconomic status, medication adherence, and post-discharge caregiver support, were not captured in the model. Despite these limitations, our findings suggest that the XGBoost model holds promise as a practical tool for early risk stratification. Future multicenter prospective studies are warranted to further validate its robustness and explore its integration into electronic health record systems.

## Conclusions

This study demonstrates that ML can effectively predict the risk of PLOS in older patients with T2DM complicated by CVD. The XGBoost model demonstrated competitive predictive performance (training AUC = 0.819; internal validation AUC = 0.753; external validation AUC = 0.728), with cerebral infarction, WBC count, and anemia identified as key predictors. However, the performance advantage over simpler models such as logistic regression was modest in validation, and we acknowledge that XGBoost showed some degree of overfitting. The model has undergone internal validation via random splitting and temporal external validation within a single institution; however, it has not yet been validated in geographically or institutionally independent cohorts. This tool shows promise for early risk stratification and resource optimization within our institutional setting, but prospective implementation and multi-center validation studies are essential before clinical adoption. Future multicenter prospective studies are warranted to further validate its robustness across diverse healthcare settings and to explore its integration into electronic health record systems for real-time clinical decision support.

## Data Availability

The raw data supporting the conclusions of this article will be made available by the authors, without undue reservation.
