# Supplementary material for: Machine learning-based prediction of prolonged length of stay in older patients with type 2 diabetes mellitus and cardiovascular disease
Source: Front Cardiovasc Med. 2026 Aug 5;13:1903579. doi: 10.3389/fcvm.2026.1903579 (PMC13486238; doi:10.3389/fcvm.2026.1903579)
Supplement: Supplementary file 1 [file Supplementaryfile1.docx]

**Table S1.** Summary of variable characteristics in this study

| **Variables** | **Type of variables** | **Form of expression** |
| --- | --- | --- |
| **Demographics** |  |  |
| Age (year) | Continuous | median (interquartile spacing) |
| Sex (n, %) | Categorical | number (proportions) |
| Smoking history (n, %) | Categorical | number (proportions) |
| Drinking history (n, %) | Categorical | number (proportions) |
| **Comorbidities** |  |  |
| Anemia (n, %) | Categorical | number (proportions) |
| COPD (n, %) | Categorical | number (proportions) |
| Cerebral infarction (n, %) | Categorical | number (proportions) |
| Osteoporosis (n, %) | Categorical | number (proportions) |
| Hypokalemia (n, %) | Categorical | number (proportions) |
| CKD (n, %) | Categorical | number (proportions) |
| **Vital signs** |  |  |
| SBP (mmHg) | Continuous | median (interquartile spacing) |
| DBP (mmHg) | Continuous | median (interquartile spacing) |
| Pulse rate (bpm) | Continuous | median (interquartile spacing) |
| **Laboratory parameters** |  |  |
| TC (mmol/l) | Continuous | median (interquartile spacing) |
| TG (mmol/l) | Continuous | median (interquartile spacing) |
| Cr (umol/l) | Continuous | median (interquartile spacing) |
| UA (umol/l) | Continuous | median (interquartile spacing) |
| LDL-C (mmol/L) | Continuous | median (interquartile spacing) |
| HDL-C (mmol/L) | Continuous | median (interquartile spacing) |
| Hemoglobin (g/L) | Continuous | median (interquartile spacing) |
| HbA1c (%) | Continuous | median (interquartile spacing) |
| FG (mmol/l) | Continuous | median (interquartile spacing) |
| WBC count (×10^9/L) | Continuous | median (interquartile spacing) |
| PLT count (×10^9/L) | Continuous | median (interquartile spacing) |
| **Composite indicators** |  |  |
| TyG | Continuous | median (interquartile spacing) |
| TC/HDL-C | Continuous | median (interquartile spacing) |
| UHR (%) | Continuous | median (interquartile spacing) |
| AIP | Continuous | median (interquartile spacing) |
| NHHR | Continuous | median (interquartile spacing) |
| GHR | Continuous | median (interquartile spacing) |
| UA/Cr | Continuous | median (interquartile spacing) |
| SHR | Continuous | median (interquartile spacing) |

Abbreviations: **COPD**, chronic obstructive pulmonary disease; **CKD**, chronic kidney disease; **SBP**, systolic blood pressure; **DBP**, diastolic blood pressure; **TC**, total cholesterol; **TG**, triglycerides; **Cr**, creatinine; **UA**, uric acid; **LDL-C**, low‑density lipoprotein cholesterol; **HDL-C**, high‑density lipoprotein cholesterol; **HbA1c**, glycated hemoglobin; **FG**, fasting glucose; **WBC**, white blood cell; **PLT**, platelet; **TyG**, triglyceride glucose; **TC/HDL-C**, total cholesterol to high-density lipoprotein cholesterol ratio; **UHR**, uric acid to high-density lipoprotein cholesterol ratio; **AIP**, atherogenic index of plasma; **NHHR**, non-high-density lipoprotein cholesterol to high-density lipoprotein cholesterol ratio; **GHR**, glycated hemoglobin to high-density lipoprotein cholesterol ratio; **UA/Cr**, uric acid to creatinine ratio; **SHR**, stress hyperglycemia ratio.

**Table S2.** Descriptive statistics of continuous variables in the training set

| **Variables** | **Total (n)** | **Mean** | **SD** | **Median** | **25 % quantile** | **75 % quantile** | **The min** | **The max** | **Missing Rate (%)** |
| --- | --- | --- | --- | --- | --- | --- | --- | --- | --- |
| Age (years) | 17,570 | 74.9 | 6.61 | 74 | 70 | 79 | 65 | 104 | 0 |
| SBP (mmHg) | 17,496 | 139.3 | 21.37 | 137 | 125 | 152 | 60 | 259 | 0.42 |
| DBP (mmHg) | 17,497 | 79.7 | 12.21 | 80 | 71 | 87 | 32 | 163 | 0.42 |
| Pulse rate (bpm) | 17,472 | 82.77 | 15.02 | 80 | 73 | 90 | 18 | 202 | 0.56 |
| TC (mmol/l) | 13,859 | 4.2 | 1.22 | 4.12 | 3.36 | 4.92 | 0.26 | 18.91 | 21.12 |
| TG (mmol/l) | 13,817 | 1.74 | 1.66 | 1.36 | 0.98 | 1.97 | 0.12 | 39.82 | 21.36 |
| Cr (umol/l) | 16,374 | 95.25 | 96.79 | 71 | 56 | 94 | 16 | 1132 | 6.81 |
| UA (umol/l) | 16,374 | 340.78 | 119.29 | 326 | 259 | 403 | 50 | 969 | 6.81 |
| LDL-C (mmol/L) | 13,772 | 2.52 | 1.02 | 2.43 | 1.75 | 3.16 | 0.24 | 9.92 | 21.62 |
| HDL-C (mmol/L) | 13,756 | 1.08 | 0.33 | 1.03 | 0.86 | 1.26 | 0.12 | 3.01 | 21.71 |
| Hemoglobin (g/L) | 16,602 | 124.31 | 20.72 | 126 | 114 | 138 | 20 | 205 | 5.51 |
| HbA1c (%) | 12,820 | 8.02 | 1.98 | 7.5 | 6.6 | 8.9 | 4 | 20.4 | 27.03 |
| FG (mmol/l) | 13,266 | 8.41 | 3.53 | 7.4 | 6.2 | 9.7 | 1.1 | 32.42 | 24.5 |
| WBC count (×10^9/L) | 16,602 | 7.56 | 3.3 | 6.8 | 5.5 | 8.7 | 0.9 | 41.9 | 5.51 |
| PLT count (×10^9/L) | 16,590 | 31.31 | 69.5 | 185 | 146 | 228 | 1 | 611 | 5.58 |

Abbreviations: **SD**, standard deviation; **SBP**, systolic blood pressure; **DBP**, diastolic blood pressure; **TC**, total cholesterol; **TG**, triglycerides; **Cr**, creatinine; **UA**, uric acid; **LDL-C**, low‑density lipoprotein cholesterol; **HDL-C**, high‑density lipoprotein cholesterol; **HbA1c**, glycated hemoglobin; **FG**, fasting glucose; **WBC**, white blood cell; **PLT**, platelet.

**Table S3.** Descriptive statistics of continuous variables in the internal validation set

| **Variables** | **Total (n)** | **Mean** | **SD** | **Median** | **25 % quantile** | **75 % quantile** | **The min** | **The max** | **Missing Rate (%)** |
| --- | --- | --- | --- | --- | --- | --- | --- | --- | --- |
| Age (years) | 7,531 | 74.8 | 6.58 | 74 | 69 | 79 | 65 | 101 | 0 |
| SBP (mmHg) | 7,498 | 138.79 | 21.1 | 137 | 125 | 151 | 65 | 239 | 0.44 |
| DBP (mmHg) | 7,497 | 79.51 | 12.39 | 79 | 71 | 87 | 30 | 167 | 0.45 |
| Pulse rate (bpm) | 7,495 | 82.51 | 15.2 | 80 | 72 | 90 | 18 | 186 | 0.48 |
| TC (mmol/l) | 5,961 | 4.19 | 1.19 | 4.11 | 3.35 | 4.9 | 0.12 | 15.33 | 20.85 |
| TG (mmol/l) | 5,953 | 1.73 | 1.66 | 1.36 | 0.97 | 1.96 | 0.25 | 43.02 | 20.95 |
| Cr (umol/l) | 7,016 | 92.33 | 88.78 | 70 | 56 | 93 | 17 | 1075 | 6.84 |
| UA (umol/l) | 7,011 | 339.46 | 119.27 | 323 | 258 | 403 | 44 | 963 | 6.9 |
| LDL-C (mmol/L) | 5,925 | 2.52 | 1.02 | 2.42 | 1.77 | 3.17 | 0.22 | 8.51 | 21.33 |
| HDL-C (mmol/L) | 5,919 | 1.08 | 0.33 | 1.04 | 0.86 | 1.25 | 0.13 | 3.9 | 21.4 |
| Hemoglobin (g/L) | 7,118 | 124.13 | 20.36 | 126 | 114 | 138 | 30 | 196 | 5.48 |
| HbA1c (%) | 5,451 | 8.04 | 1.99 | 7.5 | 6.6 | 8.9 | 4 | 18.3 | 27.62 |
| FG (mmol/l) | 5,667 | 8.43 | 3.69 | 7.4 | 6.1 | 9.6 | 0.4 | 32.7 | 24.75 |
| WBC count (×10^9/L) | 7,118 | 7.54 | 3.41 | 6.8 | 5.5 | 8.6 | 0.8 | 49.5 | 5.48 |
| PLT count (×10^9/L) | 7,115 | 191.85 | 70.57 | 183 | 145 | 228 | 1 | 617 | 5.52 |

Abbreviations: **SD**, standard deviation; **SBP**, systolic blood pressure; **DBP**, diastolic blood pressure; **TC**, total cholesterol; **TG**, triglycerides; **Cr**, creatinine; **UA**, uric acid; **LDL-C**, low‑density lipoprotein cholesterol; **HDL-C**, high‑density lipoprotein cholesterol; **HbA1c**, glycated hemoglobin; **FG**, fasting glucose; **WBC**, white blood cell; **PLT**, platelet.

**Table S4.** Descriptive statistics of continuous variables in the external validation set

| **Variables** | **Total (n)** | **Mean** | **SD** | **Median** | **25 % quantile** | **75 % quantile** | **The min** | **The max** | **Missing Rate (%)** |
| --- | --- | --- | --- | --- | --- | --- | --- | --- | --- |
| Age (years) | 2,528 | 76.07 | 6.77 | 75 | 71 | 81 | 65 | 98 | 0 |
| SBP (mmHg) | 2,524 | 137.18 | 21.22 | 136 | 123 | 150 | 69 | 238 | 0.16 |
| DBP (mmHg) | 2,524 | 78.83 | 12.51 | 78 | 71 | 86.25 | 40 | 156 | 0.16 |
| Pulse rate (bpm) | 2,525 | 82.46 | 15.29 | 80 | 72 | 91 | 41 | 173 | 0.12 |
| TC (mmol/l) | 2,071 | 4.03 | 1.3 | 3.83 | 3.17 | 4.755 | 0.45 | 14.04 | 18.08 |
| TG (mmol/l) | 2,073 | 1.78 | 2.01 | 1.35 | 0.97 | 1.98 | 0.11 | 54.8 | 18 |
| Cr (umol/l) | 2,300 | 94.79 | 89.97 | 72 | 57 | 96 | 25 | 1090 | 9.02 |
| UA (umol/l) | 2,293 | 336.91 | 119.47 | 320 | 254 | 400 | 47 | 1206 | 9.3 |
| LDL-C (mmol/L) | 2,069 | 2.35 | 1.01 | 2.21 | 1.58 | 3.02 | 0.28 | 8.1 | 18.16 |
| HDL-C (mmol/L) | 2,066 | 0.99 | 0.31 | 0.94 | 0.79 | 1.14 | 0.21 | 2.74 | 18.28 |
| Hemoglobin (g/L) | 2,358 | 124.73 | 21.54 | 127 | 114 | 138 | 32 | 189 | 6.72 |
| HbA1c (%) | 1,905 | 7.82 | 1.78 | 7.3 | 6.6 | 8.5 | 5 | 18.4 | 24.64 |
| FG (mmol/l) | 1,947 | 8.11 | 3.58 | 7 | 5.8 | 9.4 | 2.5 | 29.7 | 22.98 |
| WBC count (×10^9/L) | 2,368 | 7.56 | 3.41 | 6.7 | 5.5 | 8.6 | 0.5 | 34.5 | 6.33 |
| PLT count (×10^9/L) | 2,368 | 193.9 | 70.7 | 185 | 151 | 226 | 17 | 712 | 6.33 |

Abbreviations: **SD**, standard deviation; **SBP**, systolic blood pressure; **DBP**, diastolic blood pressure; **TC**, total cholesterol; **TG**, triglycerides; **Cr**, creatinine; **UA**, uric acid; **LDL-C**, low‑density lipoprotein cholesterol; **HDL-C**, high‑density lipoprotein cholesterol; **HbA1c**, glycated hemoglobin; **FG**, fasting glucose; **WBC**, white blood cell; **PLT**, platelet.

**Table S5.** Hyperparameter tuning ranges and final optimal values for each ML model

| **Algorithm** | **Hyperparameter** | **Search space** | **Optimal value** |
| --- | --- | --- | --- |
| XGBoost | n_estimators | {100, 200, 300, 500, 800,1000} | 300 |
|  | learning_rate | {0.001, 0.01, 0.05, 0.1, 0.2} | 0.01 |
|  | max_depth | {3, 4, 5, 6, 8, 10} | 6 |
|  | subsample | {0.6, 0.7, 0.8, 0.9, 1.0} | 0.9 |
|  | colsample_bytree | {0.6, 0.7, 0.8, 0.9, 1.0} | 0.8 |
|  | gamma | {0, 0.1, 0.2, 0.5, 1.0} | 0.2 |
|  | reg_lambda | {0.01, 0.1, 1, 10} | 0.1 |
|  | min_child_weight | {1, 3, 5, 7} | 1 |
|  | scale_pos_weight | [Calculated dynamic imbalance ratio] | imb_ratio |
| LightGBM | n_estimators | {100, 200, 300, 500, 800,1000} | 800 |
|  | learning_rate | {0.001, 0.01, 0.05, 0.1} | 0.01 |
|  | num_leaves | {15, 31, 63, 127} | 31 |
|  | max_depth | {3, 4, 5, 6, 8, 10} | 5 |
|  | min_child_samples | {10, 20, 30, 50} | 10 |
|  | subsample | {0.6, 0.7, 0.8, 0.9, 1.0} | 0.7 |
|  | reg_lambda | {0.01, 0.1, 1, 10} | 0.1 |
| RF | n_estimators | {100, 200, 300, 500, 800,1000} | 300 |
|  | criterion | {'gini', 'entropy'} | 'entropy' |
|  | max_depth | {3, 4, 5, 6, 8, 10} | 6 |
|  | min_samples_split | {2, 5, 8, 10} | 8 |
|  | min_samples_leaf | {1, 3, 5, 10} | 10 |
| LR | penalty | {'l1', 'l2', 'elasticnet'} | 'l2' |
|  | C | {0.0001, 0.001, 0.01, 0.1, 1, 10} | 0.1 |
|  | solver | {'lbfgs', 'liblinear', 'saga'} | 'saga' |
|  | max_iter | {100, 300, 500, 1000} | 500 |
| DT | criterion | {'gini', 'entropy'} | 'gini' |
|  | max_depth | {3, 4, 5, 6, 8, 10} | 5 |
|  | min_samples_split | {2, 5, 10, 20} | 5 |
|  | min_samples_leaf | {1, 3, 5, 10} | 3 |
| GBDT | n_estimators | {100, 200, 300, 500, 800,1000} | 200 |
|  | learning_rate | {0.001, 0.01, 0.05, 0.1, 0.2} | 0.2 |
|  | max_depth | {3, 4, 5, 6, 8, 10} | 3 |
|  | subsample | {0.6, 0.7, 0.8, 0.9, 1.0} | 0.9 |
|  | min_samples_split | {2, 5, 8, 10} | 5 |
| ANN | hidden_layer_sizes | {(32,), (64,), (64,32), (128,64,32)} | (64, 32) |
|  | activation | {'logistic', 'tanh', 'relu'} | 'relu' |
|  | solver | {'sgd', 'adam'} | 'adam' |
| SVM | C | {0.01, 0.1, 1.0, 10.0, 100.0} | 1 |
|  | kernel | {'linear', 'poly', 'rbf', 'sigmoid'} | 'rbf' |

Abbreviations: **XGBoost**, extreme Gradient Boosting; **LightGBM**, light gradient boosting machine; **RF**, random forest; **LR**, logistic regression; **DT**, decision tree; **GBDT**, gradient boosting decision tree; **ANN**, artificial neural network; **SVM**, support vector machine. Note: The scale_pos_weight parameter for XGBoost was set to the inverse class ratio (negative/positive) to handle class imbalance. This is an internal weighting mechanism of the XGBoost algorithm and does not involve external resampling or synthetic data generation.

**Table S6.** Baseline characteristics of participants in the external validation set

| **Variables** | **Total (N=25,28)** |
| --- | --- |
| Age (years) | 74.00 (71.00, 81.00) |
| Sex |  |
| Male | 1,168 (46.20) |
| Female | 1,360 (53.80) |
| Smoking history |  |
| No | 1,962 (77.61) |
| Yes | 566 (22.39) |
| Drinking history |  |
| No | 2,266 (89.64) |
| Yes | 262 (10.36) |
| Anemia |  |
| No | 2,096 (82.91) |
| Yes | 432 (17.09) |
| COPD |  |
| No | 2,284 (90.35) |
| Yes | 244 (9.65) |
| Cerebral infarction |  |
| No | 2,137 (84.53) |
| Yes | 391 (15.47) |
| Osteoporosis |  |
| No | 2,087 (82.56) |
| Yes | 441 (17.44) |
| Hypokalemia |  |
| No | 2,163 (85.56) |
| Yes | 365 (14.44) |
| CKD |  |
| No | 2,304 (91.14) |
| Yes | 224 (8.86) |
| SBP (mmHg) | 136.00 (123.00, 150.00) |
| DBP (mmHg) | 78.0 (71.00, 86.25) |
| Pulse rate (bpm) | 80.00 (72.00, 91.00) |
| TC (mmol/l) | 3.81 (3.17, 4.74) |
| TG (mmol/l) | 1.35 (0.97, 1.99) |
| Cr (umol/l) | 71.00 (57.00, 94.25) |
| UA (umol/l) | 320.00 (254.00, 399.00) |
| LDL-C (mmol/L) | 2.19 (1.57, 2.99) |
| HDL-C (mmol/L) | 0.94 (0.79, 1.14) |
| Hemoglobin (g/L) | 127.00 (114.00, 138.25) |
| HbA1c (%) | 7.30 (6.60, 8.50) |
| FG (mmol/l) | 7.10 (5.90, 9.60) |
| WBC count (×10^9/L) | 6.70 (5.50, 8.60) |
| PLT count (×10^9/L) | 185.00 (151.00, 226.00) |
| TyG | 9.01 (8.56, 9.52) |
| TC/HDL-C | 4.02 (3.32, 4.89) |
| UHR (%) | 14.44 (10.64, 20.11) |
| AIP | 0.52 (0.34, 0.71) |
| NHHR | 3.02 (2.32, 3.89) |
| GHR | 7.96 (6.27, 10.14) |
| UA/Cr | 4.33 (3.39, 5.34) |
| SHR | 0.79 (0.68, 0.94) |

Abbreviations: **COPD**, chronic obstructive pulmonary disease; **CKD**, chronic kidney disease; **SBP**, systolic blood pressure; **DBP**, diastolic blood pressure; **TC**, total cholesterol; **TG**, triglycerides; **Cr**, creatinine; **UA**, uric acid; **LDL-C**, low‑density lipoprotein cholesterol; **HDL-C**, high‑density lipoprotein cholesterol; **HbA1c**, glycated hemoglobin; **FG**, fasting glucose; **WBC**, white blood cell; **PLT**, platelet; **TyG**, triglyceride glucose; **TC/HDL-C**, total cholesterol to high-density lipoprotein cholesterol ratio; **UHR**, uric acid to high-density lipoprotein cholesterol ratio; **AIP**, atherogenic index of plasma; **NHHR**, non-high-density lipoprotein cholesterol to high-density lipoprotein cholesterol ratio; **GHR**, glycated hemoglobin to high-density lipoprotein cholesterol ratio; **UA/Cr**, uric acid to creatinine ratio; **SHR**, stress hyperglycemia ratio.

**Table S7.** Imputation outcomes for variables in the training set with <30% missingness

| **Variables** | **Before interpolation** | **After interpolation** | ***P* value** |
| --- | --- | --- | --- |
| SBP (mmHg) | 137.00 (125.00, 152.00) | 137.00 (125.00, 152.00) | 0.933 |
| DBP (mmHg) | 80.00 (71.00, 87.00) | 80.00 (71.00, 87.00) | 0.982 |
| Pulse rate (bpm) | 80.00 (73.00, 90.00) | 80.00 (73.00, 90.00) | 0.949 |
| TC (mmol/l) | 4.12 (3.36, 4.92) | 4.09 (3.35, 4.88) | 0.088 |
| TG (mmol/l) | 1.36 (0.98, 1.97) | 1.36 (0.97, 1.98) | 0.984 |
| Cr (umol/l) | 71.00 (56.00, 94.00) | 70.00 (56.00, 94.00) | 0.616 |
| UA (umol/l) | 326.00 (259.00, 403.00) | 325.00 (259.00, 403.00) | 0.707 |
| LDL-C (mmol/L) | 2.43 (1.75, 3.16) | 2.40 (1.74, 3.14) | 0.067 |
| HDL-C (mmol/L) | 1.03 (0.86, 1.26) | 1.03 (0.85, 1.25) | 0.064 |
| Hemoglobin (g/L) | 126.00 (114.00, 138.00) | 127.00 (114.00, 138.00) | 0.486 |
| HbA1c (%) | 7.50 (6.60, 8.90) | 7.40 (6.60, 8.80) | 0.010 |
| FG (mmol/l) | 7.40 (6.20, 9.70) | 7.50 (6.20, 9.80) | 0.004 |
| WBC count (×10^9/L) | 6.80 (5.50, 8.70) | 6.80 (5.50, 8.70) | 0.705 |
| PLT count (×10^9/L) | 185.00 (146.00, 228.00) | 185.00 (146.00, 228.00) | 0.884 |

Abbreviations: **SBP**, systolic blood pressure; **DBP**, diastolic blood pressure; **TC**, total cholesterol; **TG**, triglycerides; **Cr**, creatinine; **UA**, uric acid; **LDL-C**, low‑density lipoprotein cholesterol; **HDL-C**, high‑density lipoprotein cholesterol; **HbA1c**, glycated hemoglobin; **FG**, fasting glucose; **WBC**, white blood cell; **PLT**, platelet.

**Table S8.** Imputation outcomes for variables in the internal validation set with <30% missingness

| **Variables** | **Before interpolation** | **After interpolation** | ***P* value** |
| --- | --- | --- | --- |
| SBP (mmHg) | 137.00 (125.00, 151.00) | 137.00 (125.00, 151.00) | 0.991 |
| DBP (mmHg) | 79.00 (71.00, 87.00) | 79.00 (71.00, 87.00) | 0.962 |
| Pulse rate (bpm) | 80.00 (72.00, 90.00) | 80.00 (73.00, 90.00) | 0.920 |
| TC (mmol/l) | 4.11 (3.35, 4.90) | 4.09 (3.35, 4.89) | 0.754 |
| TG (mmol/l) | 1.36 (0.97, 1.96) | 1.36 (0.97, 1.97) | 0.862 |
| Cr (umol/l) | 70.00 (56.00, 93.00) | 70.00 (56.00, 92.00) | 0.803 |
| UA (umol/l) | 323.00 (258.00, 403.00) | 322.00 (257.00, 403.00) | 0.720 |
| LDL-C (mmol/L) | 2.42 (1.77, 3.17) | 2.42 (1.76, 3.16) | 0.814 |
| HDL-C (mmol/L) | 1.04 (0.86, 1.25) | 1.04 (0.85, 1.25) | 0.522 |
| Hemoglobin (g/L) | 126.00 (114.00, 138.00) | 126.00 (114.00, 138.00) | 0.394 |
| HbA1c (%) | 7.50 (6.60, 8.90) | 7.40 (6.60, 8.80) | 0.032 |
| FG (mmol/l) | 7.40 (6.10, 9.60) | 7.40 (6.20, 9.80) | 0.200 |
| WBC count (×10^9/L) | 6.80 (5.50, 8.60) | 6.80 (5.50, 8.60) | 0.689 |
| PLT count (×10^9/L) | 183.00 (145.00, 228.00) | 184.00 (145.00, 228.00) | 0.945 |

Abbreviations: **SBP**, systolic blood pressure; **DBP**, diastolic blood pressure; **TC**, total cholesterol; **TG**, triglycerides; **Cr**, creatinine; **UA**, uric acid; **LDL-C**, low‑density lipoprotein cholesterol; **HDL-C**, high‑density lipoprotein cholesterol; **HbA1c**, glycated hemoglobin; **FG**, fasting glucose; **WBC**, white blood cell; **PLT**, platelet.

**Table S9.** Imputation outcomes for variables in the external validation set with <30% missingness

| **Variables** | **Before interpolation** | **After interpolation** | ***P* value** |
| --- | --- | --- | --- |
| SBP (mmHg) | 136.00 (123.00, 150.00) | 136.00 (123.00, 150.00) | 0.972 |
| DBP (mmHg) | 78.00 (71.00, 86.25) | 78.0 (71.00, 86.25) | 0.989 |
| Pulse rate (bpm) | 80.00 (72.00, 91.00) | 80.00 (72.00, 91.00) | 0.982 |
| TC (mmol/l) | 3.83 (3.17, 4.76) | 3.81 (3.17, 4.74) | 0.797 |
| TG (mmol/l) | 1.35 (0.97, 1.98) | 1.35 (0.97, 1.99) | 0.928 |
| Cr (umol/l) | 72.00 (57.00, 96.0) | 71.00 (57.00, 94.25) | 0.670 |
| UA (umol/l) | 320.00 (254.00, 400.00) | 320.00 (254.00, 399.00) | 0.960 |
| LDL-C (mmol/L) | 2.21 (1.58, 3.02) | 2.19 (1.57, 2.99) | 0.552 |
| HDL-C (mmol/L) | 0.94 (0.79, 1.14) | 0.94 (0.79, 1.14) | 0.800 |
| Hemoglobin (g/L) | 127.00 (114.00, 138.00) | 127.00 (114.00, 138.25) | 0.749 |
| HbA1c (%) | 7.30 (6.60, 8.50) | 7.30 (6.60, 8.50) | 0.774 |
| FG (mmol/l) | 700 (5.80, 9.40) | 7.10 (5.90, 9.60) | 0.276 |
| WBC count (×10^9/L) | 6.70 (5.50, 8.60) | 6.70 (5.50, 8.60) | 0.669 |
| PLT count (×10^9/L) | 18500 (151.00, 226.00) | 185.00 (151.00, 226.00) | 0.908 |

Abbreviations: **SBP**, systolic blood pressure; **DBP**, diastolic blood pressure; **TC**, total cholesterol; **TG**, triglycerides; **Cr**, creatinine; **UA**, uric acid; **LDL-C**, low‑density lipoprotein cholesterol; **HDL-C**, high‑density lipoprotein cholesterol; **HbA1c**, glycated hemoglobin; **FG**, fasting glucose; **WBC**, white blood cell; **PLT**, platelet.

**Table S10.** Multicollinearity diagnostics for the 14 LASSO-selected predictors: variance inflation factor (VIF) and tolerance

| **Variables** | **VIF** | **Tolerance** |
| --- | --- | --- |
| Age (years) | 1.075 | 0.930 |
| Anemia | 1.705 | 0.587 |
| COPD | 1.087 | 0.920 |
| Cerebral infarction | 1.031 | 0.970 |
| Osteoporosis | 1.022 | 0.979 |
| CKD | 1.231 | 0.813 |
| Pulse rate (bpm) | 1.094 | 0.914 |
| HDL-C (mmol/L) | 2.777 | 0.360 |
| Hemoglobin (g/L) | 1.681 | 0.595 |
| FG (mmol/l) | 3.877 | 0.258 |
| WBC count (×10^9/L) | 1.100 | 0.909 |
| GHR | 4.089 | 0.245 |
| UA/Cr | 1.220 | 0.820 |
| SHR | 3.046 | 0.328 |

Abbreviations: **COPD**, chronic obstructive pulmonary disease; **CKD**, chronic kidney disease; **HDL-C**, high‑density lipoprotein cholesterol; **FG**, fasting glucose; **WBC**, white blood cell; **GHR**, glycated hemoglobin to high-density lipoprotein cholesterol ratio; **UA/Cr**, uric acid to creatinine ratio; **SHR**, stress hyperglycemia ratio.

**Table S11.** PR‑AUC, Brier score, calibration intercept, calibration slope, and classification threshold for eight ML models across three sets

| **Model** | **Dataset** | **PR-AUC (95% CI)** | **Brier score (95% CI)** | **Calibration intercept (95% CI)** | **Calibration slope (95% CI)** | **Threshold used** |
| --- | --- | --- | --- | --- | --- | --- |
| XGBoost | Training | 0.685 (0.672-0.698) | 0.143 (0.140–0.146) | 0.310 (0.260–0.360) | 1.393 (1.349–1.438) | 0.286 |
| XGBoost | Internal validation | 0.502 (0.480-0.526) | 0.161 (0.156–0.165) | −0.039 (−0.115 to 0.037) | 1.013 (0.955–1.077) | 0.286 |
| XGBoost | External validation | 0.424 (0.383-0.473) | 0.147 (0.138–0.154) | −0.484 (−0.606 to −0.335) | 0.889 (0.790–1.003) | 0.286 |
| GBDT | Training | 0.645 (0.632-0.659) | 0.147 (0.144–0.150) | 0.203 (0.152–0.258) | 1.241 (1.200–1.284) | 0.259 |
| GBDT | Internal validation | 0.484 (0.462-0.508) | 0.165 (0.160–0.170) | −0.157 (−0.236 to −0.087) | 0.873 (0.819–0.928) | 0.259 |
| GBDT | External validation | 0.410 (0.369-0.455) | 0.149 (0.141–0.157) | −0.568 (−0.703 to −0.445) | 0.783 (0.680–0.886) | 0.259 |
| LightGBM | Training | 0.668 (0.653-0.681) | 0.174 (0.171–0.176) | −0.984 (−1.025 to −0.942) | 1.392 (1.343–1.438) | 0.508 |
| LightGBM | Internal validation | 0.495 (0.472-0.521) | 0.196 (0.192–0.200) | −0.976 (−1.033 to −0.919) | 0.959 (0.897–1.022) | 0.508 |
| LightGBM | External validation | 0.409 (0.366-0.455) | 0.203 (0.195–0.210) | −1.303 (−1.412 to −1.209) | 0.827 (0.725–0.934) | 0.508 |
| RF | Training | 0.683 (0.671-0.696) | 0.175 (0.173–0.177) | −0.908 (−0.947 to −0.870) | 1.480 (1.434–1.530) | 0.502 |
| RF | Internal validation | 0.495 (0.474-0.519) | 0.193 (0.189–0.197) | −0.930 (−0.990 to −0.873) | 1.071 (1.005–1.137) | 0.502 |
| RF | External validation | 0.418 (0.375-0.462) | 0.196 (0.189–0.204) | −1.260 (−1.367 to −1.161) | 0.934 (0.822–1.052) | 0.502 |
| LR | Training | 0.511 (0.496-0.526) | 0.168 (0.165–0.171) | 0.005 (−0.049 to 0.063) | 1.006 (0.960–1.050) | 0.250 |
| LR | Internal validation | 0.496 (0.473-0.521) | 0.165 (0.160–0.170) | −0.074 (−0.159 to 0.014) | 0.977 (0.908–1.048) | 0.250 |
| LR | External validation | 0.412 (0.373-0.458) | 0.147 (0.140–0.155) | −0.513 (−0.665 to −0.373) | 0.916 (0.795–1.048) | 0.250 |
| DT | Training | 0.503 (0.487-0.517) | 0.205 (0.202–0.208) | −0.995 (−1.030 to −0.961) | 1.000 (0.958–1.042) | 0.568 |
| DT | Internal validation | 0.438 (0.417-0.461) | 0.211 (0.207–0.215) | −1.035 (−1.089 to −0.983) | 0.782 (0.696–0.872) | 0.568 |
| DT | External validation | 0.373 (0.335-0.418) | 0.206 (0.199–0.214) | −1.349 (−1.463 to −1.251) | 0.860 (0.754–0.979) | 0.568 |
| ANN | Training | 0.543 (0.528-0.560) | 0.161 (0.158–0.164) | −0.034 (−0.081 to 0.016) | 1.046 (1.007–1.086) | 0.242 |
| ANN | Internal validation | 0.495 (0.472-0.519) | 0.162 (0.157–0.167) | −0.152 (−0.233 to −0.073) | 0.960 (0.899–1.023) | 0.242 |
| ANN | External validation | 0.415 (0.376-0.462) | 0.149 (0.141–0.157) | −0.582 (−0.708 to −0.445) | 0.847 (0.741–0.957) | 0.242 |
| SVM | Training | 0.597 (0.582-0.613) | 0.153 (0.150–0.155) | 0.152 (0.106–0.201) | 1.206 (1.169–1.246) | 0.321 |
| SVM | Internal validation | 0.483 (0.459-0.506) | 0.162 (0.158–0.167) | −0.073 (−0.149 to 0.003) | 0.982 (0.923–1.043) | 0.321 |
| SVM | External validation | 0.371 (0.335-0.413) | 0.152 (0.144–0.160) | −0.567 (−0.706 to −0.433) | 0.800 (0.694–0.912) | 0.321 |

Abbreviations: **PR**, precision‑recall; **AUC**, area under the ROC curve; **CI**, confidence interval; **XGBoost**, extreme Gradient Boosting; **GBDT**, gradient boosting decision tree; **LightGBM**, light gradient boosting machine; **RF**, random forest; **LR**, logistic regression; **DT**, decision tree; **ANN**, artificial neural network; **SVM**, support vector machine. Note: The “Threshold used” column reports the model-specific probability cutoff derived from the training set by maximizing the Youden index. These thresholds were applied consistently to the internal and external validation sets for each respective model. All sensitivity, specificity, F1, and Youden index values reported in Tables 3–4 and Supplementary Table S12 were calculated using these thresholds.

**Table S12.** Predictive performance of eight ML models for PLOS risk in the external validation set

| **Model** | **AUC (95% CI)** | **Sensitivity (95% CI)** | **Specificity (95% CI)** | **F1 score (95% CI)** | **Youden index** |
| --- | --- | --- | --- | --- | --- |
| XGBoost | 0.728 (0.700–0.751) | 0.638 (0.596–0.677) | 0.722 (0.703–0.741) | 0.466 (0.431–0.496) | 0.360 |
| GBDT | 0.721 (0.694–0.745) | 0.654 (0.612–0.694) | 0.691 (0.671–0.710) | 0.455 (0.424–0.486) | 0.345 |
| LightGBM | 0.719 (0.697–0.745) | 0.615 (0.576–0.661) | 0.728 (0.709–0.748) | 0.457 (0.425–0.490) | 0.342 |
| RF | 0.723 (0.698–0.747) | 0.613 (0.573–0.655) | 0.739 (0.720–0.759) | 0.464 (0.431–0.497) | 0.352 |
| LR | 0.728 (0.705–0.754) | 0.675 (0.636–0.718) | 0.669 (0.649–0.690) | 0.453 (0.423–0.484) | 0.345 |
| DT | 0.717 (0.694–0.741) | 0.583 (0.543–0.625) | 0.760 (0.742–0.779) | 0.461 (0.428–0.492) | 0.343 |
| ANN | 0.719 (0.694–0.744) | 0.667 (0.628–0.709) | 0.662 (0.639–0.681) | 0.445 (0.412–0.478) | 0.329 |
| SVM | 0.707 (0.680–0.732) | 0.618 (0.574–0.659) | 0.731 (0.711–0.748) | 0.462 (0.427–0.493) | 0.350 |

Abbreviations: **AUC**, area under the ROC curve; **CI**, confidence interval; **XGBoost**, extreme Gradient Boosting; **GBDT**, gradient boosting decision tree; **LightGBM**, light gradient boosting machine; **RF**, random forest; **LR**, logistic regression; **DT**, decision tree; **ANN**, artificial neural network; **SVM**, support vector machine. All sensitivity, specificity, F1, and Youden index values were calculated using model-specific thresholds derived from the training set by maximizing the Youden index. These thresholds are reported in Supplementary Table S11.

**Table S13.** Stability of XGBoost model performance across five imputed datasets (training set)

| **Imputed dataset** | **AUC** | **Sensitivity** | **Specificity** | **F1 Score** | **Brier score** |
| --- | --- | --- | --- | --- | --- |
| Dataset 1 | 0.819 | 0.732 | 0.777 | 0.627 | 0.143 |
| Dataset 2 | 0.818 | 0.730 | 0.775 | 0.625 | 0.144 |
| Dataset 3 | 0.820 | 0.733 | 0.778 | 0.628 | 0.143 |
| Dataset 4 | 0.817 | 0.729 | 0.776 | 0.624 | 0.144 |
| Dataset 5 | 0.819 | 0.731 | 0.777 | 0.626 | 0.143 |
| Mean (SD) | 0.819 (0.001) | 0.731 (0.002) | 0.777 (0.001) | 0.626 (0.002) | 0.143 (0.001) |

Abbreviations: **AUC**, area under the ROC curve. Note: Performance metrics were calculated using the same model architecture and hyperparameters across all five imputed datasets. The minimal across-dataset variability confirms that using the first imputed dataset for model development did not materially affect the results.

**Figure S1.** Patient selection and data partitioning workflow
